# Systematic analysis of gout burden among young adults in China from 1990 to 2021: findings from the global burden of disease study 2021

**DOI:** 10.3389/fpubh.2025.1613801

**Published:** 2025-06-09

**Authors:** Yuxin Zhong, Yan Li, Chenyu Hu, Tao Tao, Liyuan Hao, Na Li, Xiaoyan Zeng, Zixin Zhang, Xiaoyu Hu

**Affiliations:** ^1^Clinical Medical College, Chengdu University of Traditional Chinese Medicine, Chengdu, China; ^2^Department of Stomach (Gastroenterology), Nantong Hospital of Traditional Chinese Medicine, Nantong, China; ^3^College of Traditional Chinese Medicine, Chongqing Medical University, Chongqing, China; ^4^Department of Infectious Diseases, Hospital of Chengdu University of Traditional Chinese Medicine, Chengdu, China

**Keywords:** gout, young adults, global burden of disease, China, ARIMA model, risk factors

## Abstract

**Background:**

Gout, a disabling inflammatory arthritis, closely linked to metabolic diseases such as hyperuricemia, obesity, hypertension, and diabetes, is increasingly prevalent among young adults in China. Understanding the burden of gout and its risk factors among young adults in China is crucial for developing effective prevention and management strategies.

**Methods:**

We analyzed data from the Global Burden of Disease Study 2021 (GBD 2021) on gout prevalence, incidence, and disability-adjusted life years (DALYs) among young adults (individuals aged 15–39 years) in China from 1990 to 2021. Joinpoint regression and the AutoRegressive Integrated Moving Average (ARIMA) model were used to assess trends and predict future burden. Summary exposure values (SEVs) were used to evaluate risk factors.

**Results:**

The study found that the burden of gout among young adults in China is significantly higher than the global average and shows a continuous upward trend. Males have a higher burden and age-standardized rates on all indicators, including prevalence, incidence, and DALYs. Joinpoint regression analysis revealed that from 1990 to 2021, the burden of gout has experienced rapid growth, stabilization, and a resurgence of acceleration since 2019. ARIMA model projects different epidemiological trends for gout across 2022–2036, with age-standardized prevalence rate (ASPR) and age-standardized incidence rate (ASIR) declining while age-standardized DALYs rate (ASDR) gradually rise. Gender-specific disparities persist. Females demonstrate a predictable rise in ASPR, ASIR and ASDR. Despite males facing higher baseline burdens and accelerating ASDR growth, their ASPR and ASIR exhibit downward trends. A significant rise in gout risk factors among Chinese aged 15–39 from 1990 to 2021 highlights the need to face the upcoming gout burden and carry out targeted measures for this population.

**Conclusion:**

The rising burden of gout among young Chinese adults demands immediate gender- and age-specific public health action. Targeted interventions focusing on modifiable risk factors such as dietary habits and lifestyle are crucial to reduce the impact of gout on young adults.

## 1 Introduction

Gout, a type of inflammatory arthritis characterized by recurrent acute joint inflammation, arises from the deposition of monosodium urate crystals in joints and tissues due to hyperuricemia (1, 2). The global burden of gout continues to increase, with the older adult population being the primary contributors to its growth (3, 4). Nonetheless, a significant and growing trend in young adults is emerging, which is frequently neglected in public health considerations (5). This shift in the epidemiology of gout has significant impact for public health and healthcare systems.

Current research on gout is marked by several limitations, including an age bias favoring the older adult, resulting in a poor understanding of the situation in younger populations (6). Additionally, the lack of age-standardized rates (ASRs) for specific age groups may introduce statistical biases due to varying population distributions (7). Geographically, studies often miss specific analyses of the disease burden toward individual countries, particularly where gout’s impact is pronounced, such as in the United States, China, and Japan (8). Furthermore, the Global Burden of Disease database provides only a few risk factors of gout, like kidney dysfunction and obesity, does not capture the full spectrum of risks associated with gout (9).

China, with its large population and rapidly developing economic landscape, offers a unique context for studying the burden of gout. Changes in dietary habits, lifestyle, and increasing obesity rates are contributing to the increasing prevalence of gout (10), and these risk factors are all on the rise among young adults in China. This study aims to systematically analyze the burden of gout among young adults in China, focusing on the 15–39 age group. We will quantify the current burden and compare it with the global level. Furthermore, we will examine the temporal trends in gout burden and analyze the key modifiable risk factors contributing to its growing prevalence. By achieving these objectives, we aim to provide insights into the shifting epidemiology of gout in China and facilitate the development of evidence-based interventions to tackle this growing public health challenge.

## 2 Methods

### 2.1 Study design and data source

This study employed an observational design using data from the Global Burden of Disease Study 2021 (GBD 2021) (11). The GBD Study provides comprehensive estimates of disease burden, including prevalence, incidence, and disability-adjusted life years (DALYs) and their 95% uncertainty intervals (UI), by age, sex, and year (12). The data provided in the GBD 2021 were modeled and estimated by DisMod-MR 2.1 and Spatiotemporal Gaussian Process Regression (ST-GPR) (12). The reference case definition of gout in GBD was based on the criteria of the 1977 American Rheumatism Association (ACR) (9). We focused on individuals aged 15 to 39 years in China and analyzed the data by age and sex to assess the specific burden of gout within this demographic.

### 2.2 Data analysis

Data Extraction: We extracted data on gout prevalence, incidence, DALYs and summary exposure values (SEVs) for individuals aged 15 to 39 years from the GBD 2021 (11).

### 2.3 Age-standardization

To obtain standardized measures of the burden of gout, we first calculated the standard population ratios based on the world population data from GBD 2021 (11). The formula for the standard population ratio is as follows:

$$\text{Standard Population Ratio}=\frac{\begin{array}{c} \text{Number of People in Each}\;\\ \mathrm{Age}\;\text{Group of the World Population} \end{array}}{\text{Total World Population}}$$

The calculated World Standard Population ratios are detailed in Supplementary material 1. Subsequently, we calculated the ASRs using the ASR formula (7): $\mathrm{ASR}=\sum_{i}(r_{i}\cdot w_{i})$ . Where *r_i_* represents the actual rate in the *i*th age group of the target population, and *w_i_* is the proportion of the *i*th age group in the standard population.

### 2.4 Trend analysis

To quantify the temporal trends of incidence rate, prevalence rate, and DALY rate, we employed the Estimated Annual Percentage Change (EAPC) as our analytical approach (13). This method was chosen for its effectiveness in assessing long-term trends by accounting for exponential changes over time. The EAPC was calculated using the formula $\text{EAPC}=100\times (e^{\beta }-1)$ , where “*β*” is the regression coefficient of the time variable in the log-linear model: $\ln(r_{t})=\alpha +\mathrm{\beta t}$ . Firstly, the natural logarithm transformation of the annual rates ln(*r_t_*) was performed to linearize the trend. Subsequently, a linear regression model was fitted with ln(*r_t_*) as the dependent variable and calendar year (*t*) as the independent variable. The slope coefficient (*β*) obtained from this regression analysis represents the average annual change in log-transformed rates. Its 95% confidence interval (CI) was derived from the results of the regression analysis. If the EAPC point estimate and its 95% CI are greater than zero, this indicates an increasing trend; conversely, if both the point estimate and the 95% CI are less than zero, it suggests a declining trend. A *p*-value < 0.05 was considered statistically significant.

### 2.5 Joinpoint regression

We conducted joinpoint regression analysis to identify statistically significant changes in the direction or magnitude of the trends in ASRs over time (14). This technique allows us to detect potential inflection points and evaluate the temporal dynamics of the disease burden. The analyses were performed using the Joinpoint Regression Program (version 5.3.0), which is accessible at https://surveillance.cancer.gov/joinpoint/.

### 2.6 ARIMA modeling

The AutoRegressive Integrated Moving Average (ARIMA) model was fitted to the time-series data for each metric (prevalence, incidence, and DALYs) using the forecast package (version 8.23.0) in R (15). We utilized the ARIMA model to forecast the future trends in ASRs over the next 15 years. Stationarity was assessed through an automated procedure within the auto.arima function forecast package (version 8.23.0) (16), which combines augmented Dickey-Fuller (ADF) and Kwiatkowski-Phillips-Schmidt-Shin (KPSS) tests. The differencing order (*d*) was determined by iterative differencing until the KPSS test indicated stationarity (*p* > 0.05). Optimal autoregressive (*p*) and moving average (*q*) orders were selected through exhaustive search minimizing the corrected Akaike Information Criterion (AICc), incorporating a maximum allowed order constraint (*p* + *q* ≤ 5) to prevent overparameterization. The final model structure was identified as ARIMA (*p*, *d*, *q*) through this process. The normality of the model residuals was assessed using Q-Q plots, ACF plots, and PACF plots. The Ljung-Box test for white noise was applied to evaluate whether the residuals exhibited serial correlation.

### 2.7 Summary exposure value analysis

We conducted a SEV analysis to assess the level of exposure to risk factors for gout in the study population (17). SEVs are assessed on a scale ranging from 0 to 100, where a score of 100 indicates that the entire population is exposed to the highest level of risk, while a score of 0 signifies that the entire population is at the lowest level of risk. In GBD 2021, 73 risk factors categorized into 4 levels of the study cohort were provided, which were further classified into three types: metabolic, environmental, and behavioral risks. From these, we selected 9 main risk factors closely related to gout for analysis. We listed gout-related SEV data of the 15–39 age group to assess the impact of each risk factor.

### 2.8 Statistical procedures

Statistical analyses were conducted using R version 4.4. Data manipulation and visualization were performed using the dplyr and ggplot2 packages (18, 19).

### 2.9 Ethical considerations

This study used de-identified, publicly available data from the GBD Study, and did not require ethical approval (20).

## 3 Results

### 3.1 The overview of the disease burden of gout

In 2021, China recorded 1,434,359 prevalent cases of gout (95% UI: 959,390–2,001,763) among individuals aged 15–39 years, with an age-standardized prevalence rate (ASPR) of 262.73 per 100,000 population (95% UI: 165.28–383.32). The EAPC in ASPR from 1990 to 2021 was 0.73 (95% CI: 0.66–0.81). Similarly, the total number of incident cases reached 363,759 (95% UI: 245,080–499,881), corresponding to an age-standardized incidence rate (ASIR) of 68.33 per 100,000 population (95% UI: 40.19–103.80) and an EAPC of 0.72 (95% CI: 0.65–0.80). Regarding disability burden, China reported 48,394 DALYs (95% UI: 27,840–75,401) in 2021, with an age-standardized DALYs rate (ASDR) of 8.87 per 100,000 population (95% UI: 4.72–14.41) and an EAPC of 0.75 (95% CI: 0.67–0.82). These metrics collectively indicate that the disease burden of gout among individuals aged 15–39 years in China was significantly higher than the global average in 2021 (6), with all indicators demonstrating a progressive upward trend since 1990. This suggests an escalating impact of gout on younger populations. Detailed gender- and age-stratified data are presented in Table 1.

**Table 1:** The burden of gout among the Chinese population aged 15–39 in 2021 and its EAPC from 1990 to 2021.

Groups	Prevalence			Incidence			DALYs		
	Number (95% UI)	Rate* per 100,000 (95% UI)	EAPC (95% CI)	Number (95% UI)	Rate* per 100,000 (95% UI)	EAPC (95% CI)	Number (95% UI)	Rate* per 100,000 (95% UI)	EAPC (95% CI)
Both	1,434,359 (959,390, 2,001,763)	262.73 (165.28, 383.32)	0.73 (0.66, 0.81)	363,759 (245,080, 499,881)	68.33 (40.19, 103.8)	0.72 (0.65, 0.8)	48,394 (27,840, 75,401)	8.87 (4.72, 14.41)	0.75 (0.67, 0.82)
Sex group									
Male	1,177,127 (796,088, 1,616,146)	416.46 (264.2, 599.9)	0.81 (0.71, 0.92)	298,776 (202,249, 413,578)	108.15 (64.51, 162.82)	0.8 (0.7, 0.9)	39,711 (22,870, 61,287)	14.06 (7.51, 22.69)	0.82 (0.72, 0.93)
Female	257,232 (160,249, 380,824)	98.07 (56.7, 150.63)	0.55 (0.49, 0.61)	64,983 (42,130, 94,310)	25.42 (14.05, 40.78)	0.52 (0.46, 0.59)	8,684 (4,684, 14,066)	3.31 (1.64, 5.6)	0.56 (0.5, 0.62)
Age group									
15–19 years	3,657 (1,252, 7,861)	4.9 (1.68, 10.53)	0.33 (0.32, 0.35)	2,547 (895, 5,294)	3.41 (1.2, 7.09)	0.33 (0.31, 0.34)	128 (41, 291)	0.17 (0.05, 0.39)	0.27 (0.2, 0.33)
20–24 years	37,495 (22,067, 57,047)	51.24 (30.16, 77.96)	0.54 (0.5, 0.59)	19,045 (11,292, 27,366)	26.03 (15.43, 37.4)	0.63 (0.57, 0.69)	1,316 (671, 2,200)	1.8 (0.92, 3.01)	0.54 (0.5, 0.59)
25–29 years	169,723 (99,972, 248,333)	196.25 (115.6, 287.15)	0.63 (0.57, 0.69)	55,282 (29,932, 82,820)	63.92 (34.61, 95.77)	0.64 (0.58, 0.7)	5,841 (2,838, 9,476)	6.75 (3.28, 10.96)	0.65 (0.59, 0.71)
30–34 years	498,793 (306,565, 736,625)	411.7 (253.04, 608.01)	0.71 (0.62, 0.79)	127,677 (84,988, 176,577)	105.38 (70.15, 145.75)	0.71 (0.63, 0.79)	16,938 (8,398, 27,566)	13.98 (6.93, 22.75)	0.72 (0.63, 0.8)
35–39 years	724,690 (475,486, 1,040,410)	683.91 (448.73, 981.86)	0.8 (0.72, 0.89)	159,209 (88,505, 259,886)	150.25 (83.52, 245.26)	0.8 (0.71, 0.88)	24,171 (13,868, 38,998)	22.81 (13.09, 36.8)	0.81 (0.72, 0.9)

*The rates provided for Both, Male, and Female are age-standardized rates, while the rates 16 provided for each age group are crude rates derived from the raw data.

### 3.2 Comparison between China and global trends in gout burden (1990–2021)

#### 3.2.1 Comparison of case numbers

From 1990 to 2021, the number of prevalent cases of gout among individuals aged 15–39 years in China increased by 31.9%, rising from 1,087,865 (95% UI: 721,033–1,507,148) to 1,434,359 (95% UI: 959,390–2,001,763). A transient decline occurred after 2005, followed by resurgence post-2015. In contrast, the global prevalent cases surged by 66.1%, escalating from 3,057,089 (95% UI: 2,025,036–4,291,701) to 5,077,197 (95% UI: 3,429,871–7,110,707).

For incident cases, China reported a 25.5% increase, from 289,708 cases (95% UI: 192,893–391,896) in 1990 to 363,759 cases (95% UI: 245,080–499,881) in 2021, peaking around 2005 and stabilizing after 2015. Globally, incident cases grew by 62.1%, increasing from 800,398 (95% UI: 527,205–1,100,335) to 1,296,983 (95% UI: 871,199–1,780,286).

Regarding DALYs, China showed a 31.7% rise, from 36,728 (95% UI: 21,169–57,136) to 48,394 (95% UI: 27,840–75,401), with decelerated growth after 2004. The global increase was substantially higher at 65.5%, from 103,063 (95% UI: 59,239–159,757) to 170,600 (95% UI: 100,296–263,668).

**Figure 1:**
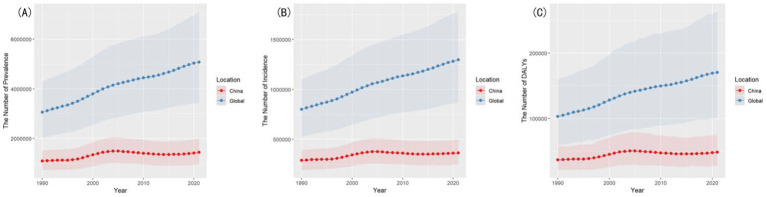
Comparison of case numbers between China and the global level. **(A)** Comparison of prevalent cases. **(B)** Comparison of incident cases. **(C)** Comparison of DALYs.

A detailed comparison of case numbers between China and the global level is presented in Figure 1.

#### 3.2.2 Comparison of age-standardized rates

China consistently exhibited higher ASRs than the global average, with steeper growth trajectories. The ASPR in China rose from 223.67 (95% UI: 139.43–325.21) to 262.73 (95% UI: 165.28–383.32), while globally, the rate increased modestly from 156.09 (95% UI: 96.49–229.06) to 170.68 (95% UI: 107.09–249.56).

Similarly, China’s ASIR climbed from 58.34 (95% UI: 34.50–88.12) to 68.33 (95% UI: 40.19–103.80), compared to the global ASIR increase from 40.08 (95% UI: 23.22–61.32) to 43.60 (95% UI: 25.44–66.34).

For ASDR, China’s rate grew from 7.55 (95% UI: 4.00–12.32) to 8.87 (95% UI: 4.72–14.41), surpassing the global trend of 5.25 (95% UI: 2.82–8.52) to 5.74 (95% UI: 3.10–9.26).

**Figure 2:**
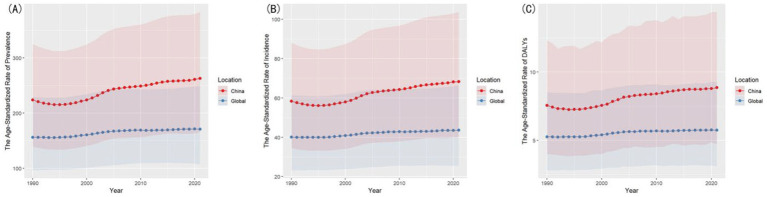
Comparison of ASRs between China and the global level. **(A)** Comparison of ASPR. **(B)** Comparison of ASIR. **(C)** Comparison of ASDR.

The comparison of ASRs between China and the global level are shown in Figure 2.

#### 3.2.3 Comparison of EAPC

The crude rate of China’s 15–39-year-old cohort increased rapidly from 1990 to 2021. Prevalence rate increased with an EAPC of 1.34 (95% CI: 1.19–1.49; *p* < 0.001; Rank 28/204). Incidence rate rose at 1.23 (95% CI: 1.14–1.33; *p* < 0.001; Rank 29/204), and DALYs rate escalated with an EAPC of 1.35 (95% CI: 1.20–1.49; *p* < 0.001; Rank 27/204).

The ranking of EAPC across 204 countries and regions is detailed in Supplementary material 2.

### 3.3 Gender and age disparities in the disease burden of gout in China

#### 3.3.1 Overview of gender disparities in 2021

In 2021, significant gender disparities in the burden of gout were observed among individuals aged 15–39 years in China. Table 2 shows that males exhibited markedly higher age-standardized rates than females across all metrics, with disparities widening progressively with age.

**Table 2:** Changes in male and female from the 15–19 age group to the 35–39 age group.

Metrics (Crude rate)	Male (95% UI)		Female (95% UI)	
Age group	15–19 years	35–39 years	15–19 years	35–39 years
Prevalence rate	6.57 (2.27, 13.99)	1,094.44 (719.18, 1,557.69)	2.96 (0.67, 6.22)	251.34 (156.30, 374.97)
Incidence rate	4.57 (1.60, 9.63)	239.86 (135.37, 388.93)	2.07 (0.47, 4.41)	55.83 (30.22, 94.29)
DALYs rate	0.23 (0.07, 0.50)	36.51 (20.88, 58.55)	0.10 (0.02, 0.24)	8.38 (4.58, 13.80)

During adolescence (15–19 years), males exhibited 2.22-fold, 2.21-fold, and 2.30-fold higher crude prevalence, incidence, and DALYs rates compared to females, indicating early-onset gender disparities in gout burden.

In late adulthood (35–39 years), gender disparities in crude rates intensified markedly. Males exhibited 4.35-fold, 4.30-fold, and 4.36-fold higher crude prevalence, incidence, and DALYs rates compared to females. The widening gender disparities reflect a more pronounced exposure to metabolic risk factors and behavioral contributors among males as they age.

**Figure 3:**
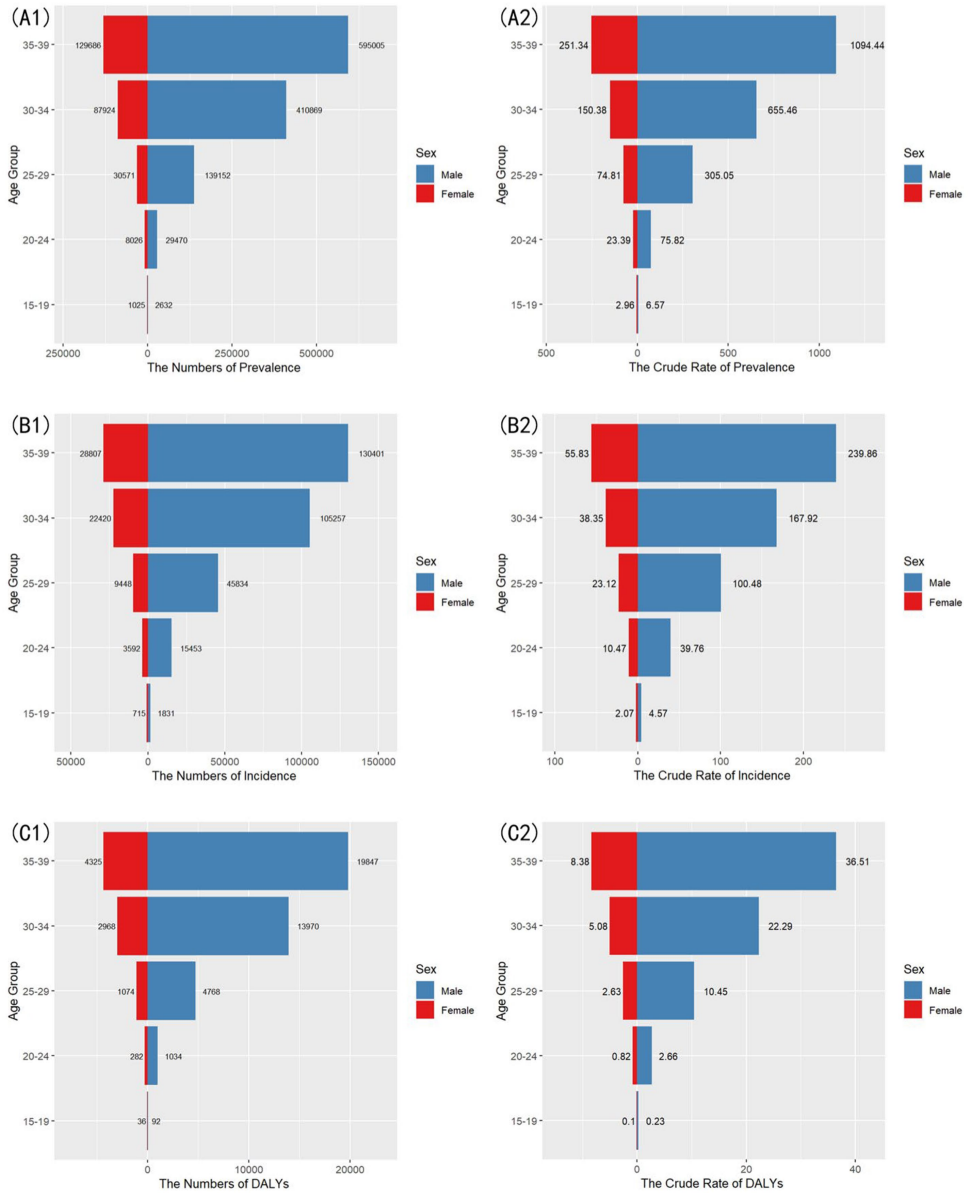
The bilateral charts illustrating the number of cases and crude rates of gout burden by sex and age in China (2021). **(A1,A2)** Comparison of prevalent cases and crude prevalence rates. **(B1,B2)** Comparison of incident cases and crude incidence rates. **(C1,C2)** Comparison of DALYs and crude DALYs rates.

The bilateral charts illustrating the number of cases and crude rates of gout burden by sex and age in China (2021) are detailed in Figure 3.

#### 3.3.2 Gender disparities in gout burden in China from 1990 to 2021

Male prevalent cases increased by 32.5% from 888,451 (95% UI: 599,765–1,213,921) to 1,177,127 (95% UI: 796,088–1,616,146), with a 17.8% rise in ASPR from 353.3 (95% UI: 221.5–510.6) to 416.5 (95% UI: 264.2–599.9). Female cases rose 29.0% from 199,414 (95% UI: 124,522–291,723) to 257,232 (95% UI: 160,249–380,824), with a 16.2% ASPR increase from 84.4 (95% UI: 49.0–129.3) to 98.1 (95% UI: 56.7–150.6).

Male incident cases grew 26.2% from 236,793 (95% UI: 161,069–317,036) to 298,776 (95% UI: 202,249–413,578), with a U-shaped ASIR which declined from 92.3 (95% UI: 55.1–138.4) in 1990 to 87.4 (95% UI: 52.2–130.7) in 1995, then rebounded sharply to 108.2 (95% UI: 64.5–162.8) in 2021. Female cases saw a 22.8% increase from 52,915 (95% UI: 33,571–74,772) to 64,983 (95% UI: 42,130–94,310) and a gradual ASIR rise from 21.9 (95% UI: 12.1–35.0) to 25.4 (95% UI: 14.1–40.8).

Male DALYs rose 32.5% from 29,958 (95% UI: 17,381–46,468) to 39,711 (95% UI: 22,870–61,287) with an 18.1% rise in ASDR from 11.9 (95% UI: 6.4–19.3) to 14.1 (95% UI: 7.5–22.7). Female DALYs rose 28.3% from 6,769 (95% UI: 3,527–11,158) to 8,684 (95% UI: 4,684–14,066), with a 16.1% ASDR rise from 2.85 (95% UI: 1.40–4.84) to 3.31 (95% UI: 1.64–5.60).

**Figure 4:**
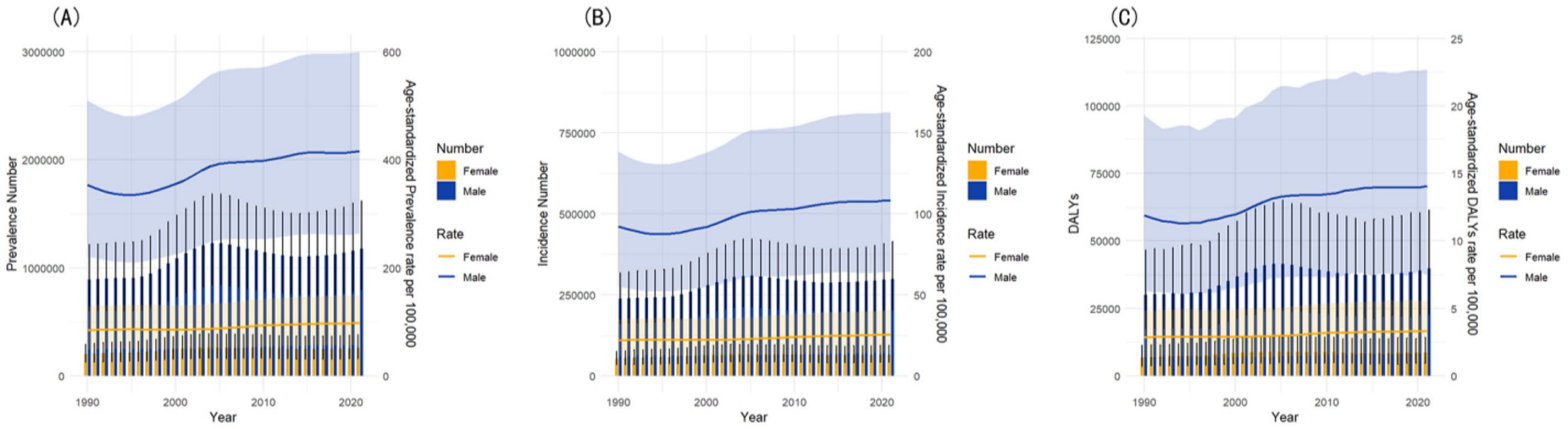
The gender disparities in gout burden in China from 1990 to 2021. **(A)** Comparison of prevalent cases and ASPR. **(B)** Comparison of incident cases and ASIR. **(C)** Comparison of DALYs and ASDR.

Overall, males consistently bore a higher gout burden and a faster growth speed in all metrics. The gender disparities in gout burden in China from 1990 to 2021 are shown by the dual-axis chart in Figure 4.

### 3.4 Joinpoint regression analysis of age-standardized rates

The ASPR initially declined significantly from 1990 to 1995 with an annual percentage change (APC) of −0.44 (95% CI: −0.55 to −0.34, *p* < 0.05), followed by sustained growth with fluctuations in acceleration rates. The most rapid increase occurred between 2000 and 2005 with an APC of 1.36 (95% CI: 1.21–1.51, *p* < 0.05). After 2019, it showed a surge of acceleration with an APC of 0.87 (95% CI: 0.39–1.34, *p* < 0.05), suggesting emerging risk factors requiring public health attention. The overall average annual percentage change (AAPC) over the study period was 0.49 (95% CI: 0.43–0.55, *p* < 0.05), indicating a significant long-term upward trend.

The ASIR declined significantly from 1990 to 1995 with an APC of −0.48 (95% CI: −0.57 to −0.39, *p* < 0.05), followed by sustained growth with accelerated phases. The most rapid increase occurred between 2001 and 2005 with an APC of 1.37 (95% CI: 1.24–1.51, *p* < 0.05). After 2019, it showed a surge of acceleration with an APC of 0.82 (95% CI: 0.39–1.25, *p* < 0.05), indicating emergent risk exposures requiring targeted interventions. The AAPC was 0.50 (95% CI: 0.45–0.56, *p* < 0.05), reflecting a significant long-term upward trend.

The ASDR declined significantly from 1990 to 1995 with an APC of −0.67 (95% CI: −1.11 to −0.22, *p* < 0.05), followed by sustained growth. A notable acceleration occurred during 2000–2004 with an APC of 1.56 (95% CI: 0.85–2.27, *p* < 0.05). The AAPC was 0.47 (95% CI: 0.35–0.59, *p* < 0.05), indicating a long-term increase in disease burden.

**Figure 5:**
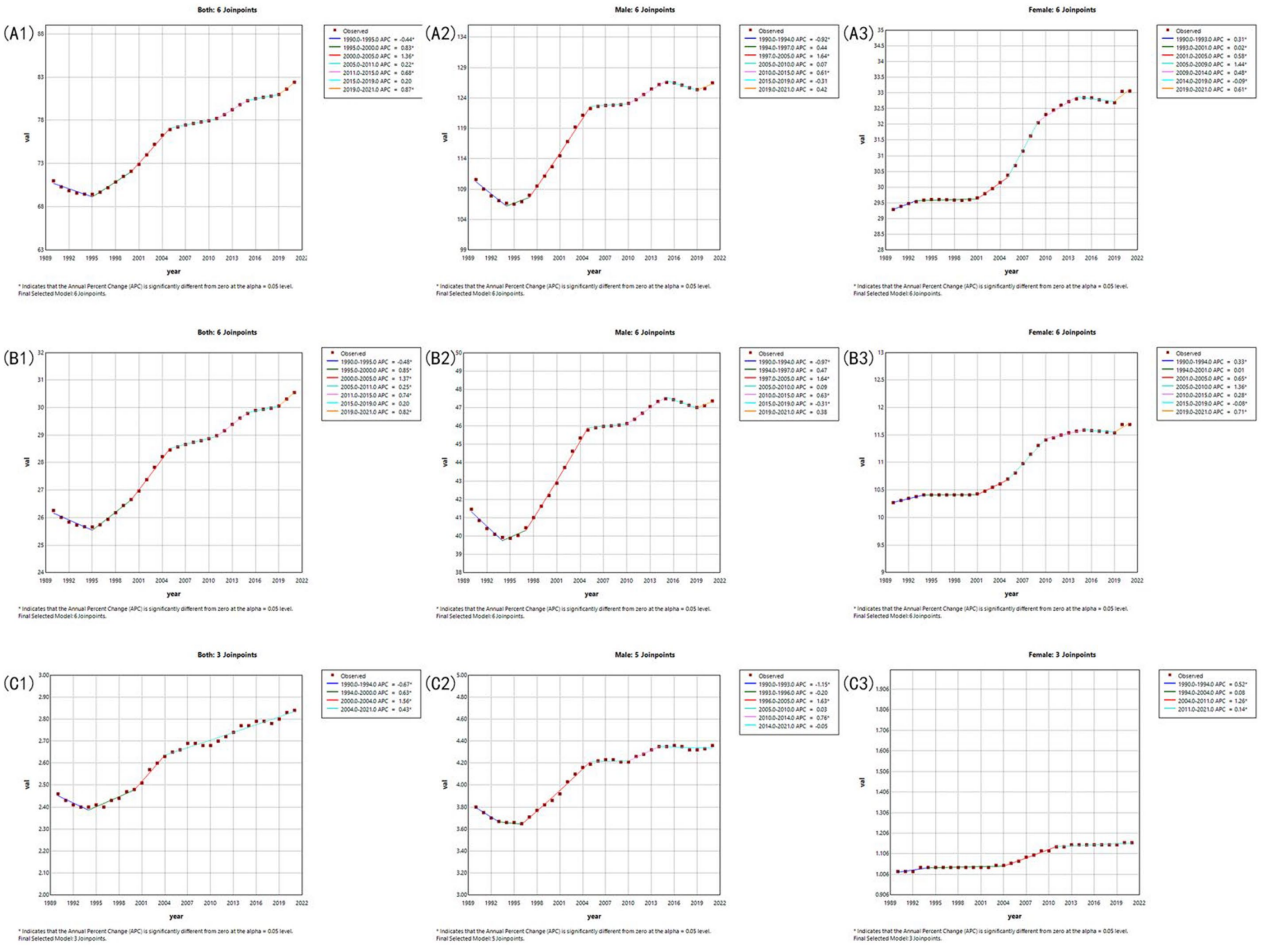
Joinpoint regression analysis of age-standardized rates. **(A1,A2,A3)** Joinpoint regression of ASPR for both, male and female. **(B1,B2,B3)** Joinpoint regression of ASIR for both, male and female. **(C1,C2,C3)** Joinpoint regression of ASDR for both, male and female.

The detailed APCs for each sex group are shown by table in Supplementary material 3 and line chart in Figure 5.

### 3.5 ARIMA model for long-term prediction of gout epidemiology (2022–2036)

#### 3.5.1 ASPR

The ASPR is expected to demonstrate a pattern of initial stabilization followed by a gradual downward trend. The forecast peaks marginally at 264.75 (95% CI: 262.19–267.31) in 2023 before entering a consistent decline phase. From 2024 (264.53, 95% CI: 259.21–269.85), the values steadily decrease to 263.80 (95% CI: 219.85–307.74) by 2036.

The male ASPR is expected to follow a short-term rise followed by gradual decline. Forecast values peak marginally at 419.85 (95% CI: 403.86–435.83) in 2025 before transitioning into a prolonged downward trajectory. From 2026 (419.91, 95% CI: 396.90–442.93), values decrease consistently, stabilizing at 418.67 (95% CI: 333.62–503.73) by 2036. The female ASPR is projected to demonstrate sustained gradual growth over the forecast period. Starting at 98.33 (95% CI: 97.90–98.76) in 2022, the values rise steadily, reaching 100.36 (95% CI: 90.07–110.65) by 2036.

#### 3.5.2 ASIR

The ASIR is projected to demonstrate a persistent downward trend with expanding uncertainty. Starting at 68.27 (95% CI: 67.97–68.57) in 2022, forecast values decline steadily to 66.40 (95% CI: 55.43–77.37) by 2036.

The male ASIR exhibits a consistent long-term decline, decreasing from 107.74 (95% CI: 107.22–108.27) in 2022 to 103.40 (95% CI: 82.56–124.25) by 2036. In contrast, the female ASIR demonstrates stable gradual growth, rising from 25.48 (95% CI: 25.38–25.58) in 2022 to 26.32 (95% CI: 22.63–30.01) by 2036.

#### 3.5.3 ASDR

The ASDR is expected to exhibit a gradual upward trajectory. Forecast values increase from 8.91 (95% CI: 8.84–8.98) in 2022 to 9.04 (95% CI: 7.58–10.50) by 2036.

The male ASDR shows an accelerating upward trend. Values climb from 14.15 (95% CI: 14.04–14.27) in 2022 to 14.73 (95% CI: 11.64–17.83) by 2036. The female ASDR displays stable incremental growth with predictable patterns. Values rise consistently from 3.32 (95% CI: 3.29–3.35) in 2022 to 3.52 (95% CI: 3.33–3.72) by 2036.

**Figure 6:**
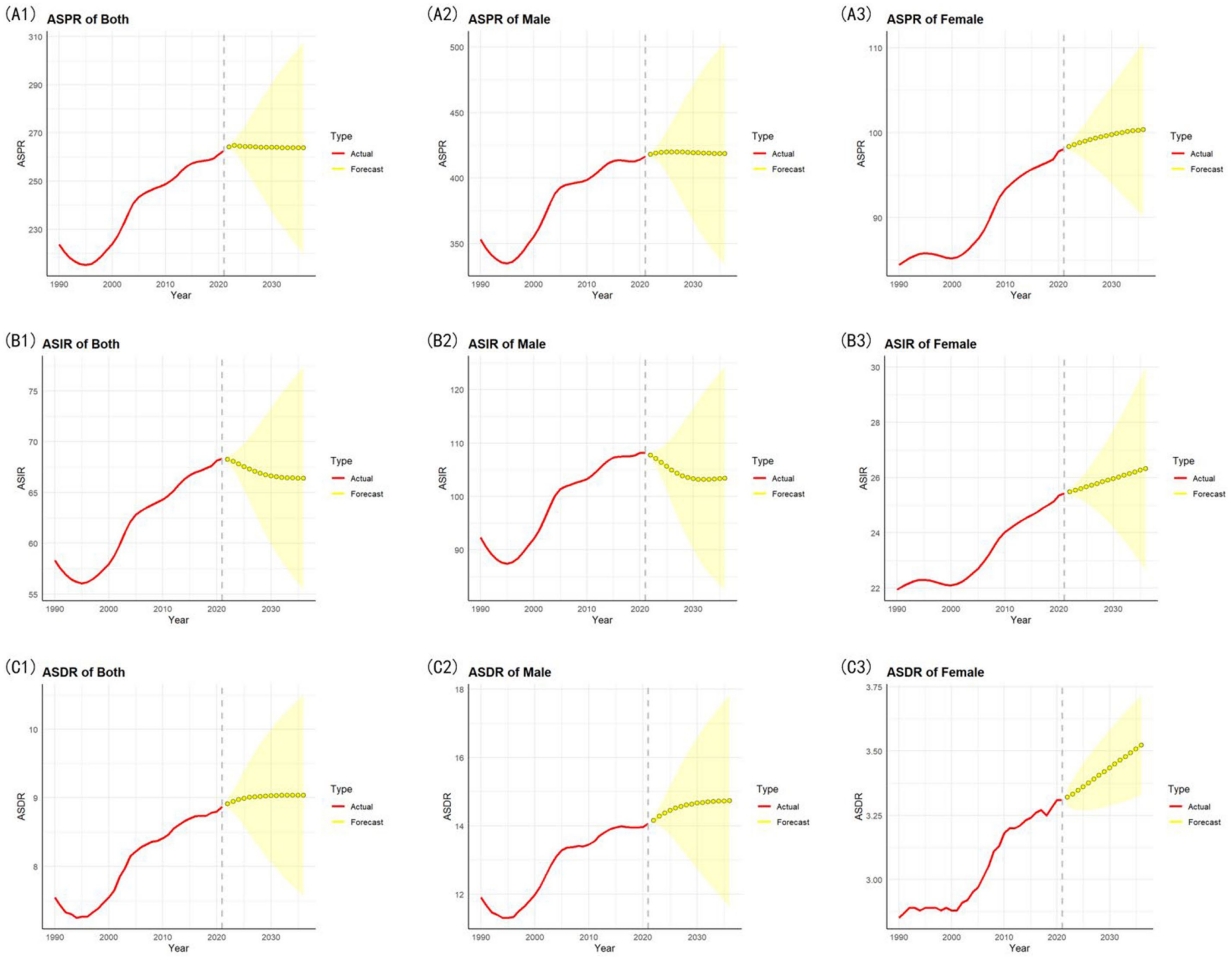
The line chart of the ARIMA model for each metric and each each sex group. **(A1,A2,A3)** ARIMA model of ASPR for both, male and female. **(B1,B2,B3)** ARIMA model of ASIR for both, male and female. **(C1,C2,C3)** ARIMA model of ASDR for both, male and female.

Figure 6 shows the line chart of the ARIMA model for each metric and each sex group. The differencing order and the selected (*p*, *d*, *q*) parameters determined by auto.arima, along with the ACF, PACF plots, QQ plot, and Ljung-Box test results are detailed in Supplementary material 4. Sensitivity analysis of the ARIMA model is detailed in Supplementary material 5.

### 3.6 Summary exposure value of risk factors for gout

We selected 9 representative risk factors for gout from the 73 risk factors provided in GBD 2021 to assess the exposure of the study population to these gout-related risks. Hypertension, high fasting glucose, high body-mass index (BMI), high alcohol use, high processed meat intake, high sugar-sweetened beverages intake, physical inactivity, chewing tobacco, and kidney dysfunction were selected to illustrate the changes of the risk factors of gout from 1990 to 2021.

Hypertension grew more prominently in males, rising from 16.79 (95% UI: 4.82–37.26) to 32.80 (95% UI: 12.54–55.74), compared to female increases from 11.88 (95% UI: 1.68–30.16) to 21.37 (95% UI: 5.30–46.36). High fasting glucose demonstrated a significant upward trend in both sexes. Among males, exposure increased from 14.56 (95% UI: 10.11–20.65) in 1990 to 22.77 (95% UI: 16.40–29.93) in 2021. Similarly, females experienced a rise from 10.94 (95% UI: 7.19–15.40) to 16.98 (95% UI: 11.21–22.90), reflecting a parallel escalation. High BMI showed substantial escalation. Females increased from 6.38 (95% UI: 5.03–8.42) in 1990 to 15.13 (95% UI: 13.30–18.38) in 2021. Males surged from 7.36 (95% UI: 5.99–9.41) to 18.36 (95% UI: 16.09–21.91). High alcohol use remained significantly higher in males, with values ascending from 12.98 (95% UI: 8.79–22.40) in 1990 to 16.54 (95% UI: 11.76–26.31) in 2021. Female alcohol exposure increased modestly from 3.72 (95% UI: 2.11–7.94) to 5.11 (95% UI: 3.10–9.64). High processed meat intake demonstrated a continuous upward trend. Among females, exposure increased from 2.61 (95% UI: 1.52–3.30) in 1990 to 6.87 (95% UI: 4.71–8.55) in 2021. Among males, values rose from 2.42 (95% UI: 1.50–3.06) to 5.82 (95% UI: 3.97–7.27) over the same period. High sugar-sweetened beverage intake exhibited rapid growth. Female exposure increased from 1.33 (95% UI: 0.90–1.90) in 1990 to 7.03 (95% UI: 4.63–9.92) in 2021, while male exposure rose from 1.20 (95% UI: 0.85–1.71) to 6.28 (95% UI: 4.04–8.84). Physical inactivity showed a gradual increase over the study period. Male exposure rose from 4.71 (95% UI: 1.79–9.82) to 6.40 (95% UI: 2.39–12.17), while female exposure climbed from 11.25 (95% UI: 5.46–18.78) to 13.91 (95% UI: 7.03–22.04). Chewing tobacco exhibited a notable gender disparity. Male exposure remained consistently higher than female, with values increasing from 0.96 (95% UI: 0.55–1.57) in 1990 to 1.16 (95% UI: 0.66–1.90) in 2021. Female exposure showed a gradual rise from 0.37 (95% UI: 0.17–0.69) to 0.49 (95% UI: 0.23–0.94) over the same period. Kidney dysfunction declined slightly in both groups. Females decreased from 0.87 (95% UI: 0.63–1.30) to 0.73 (95% UI: 0.49–1.14), and males from 0.77 (95% UI: 0.55–1.14) to 0.65 (95% UI: 0.44–1.00).

**Figure 7:**
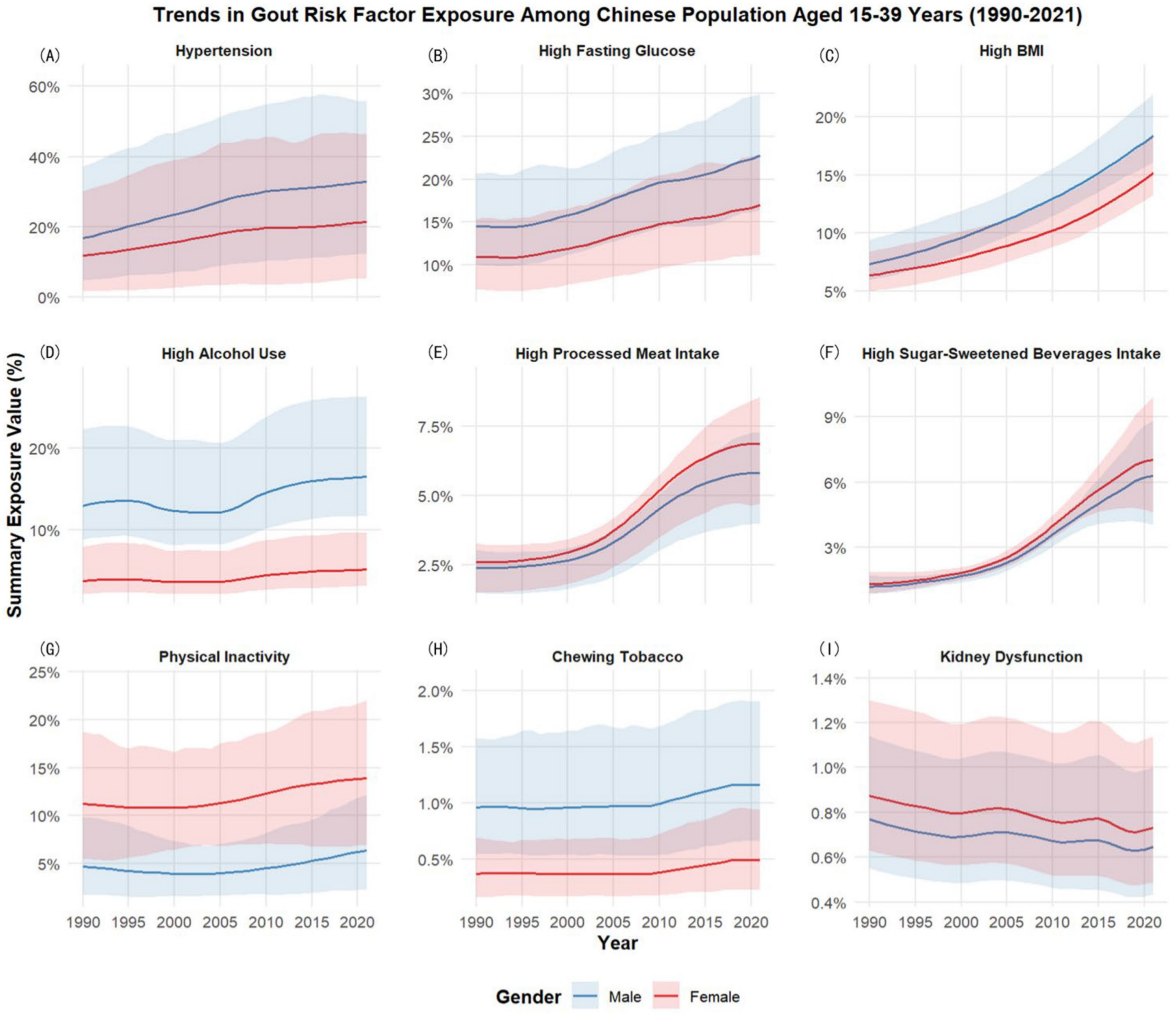
The temporal trends of exposure to gout risk factors among young adults in China from 1990 to 2021. **(A)** Hypertension; **(B)** High fasting glucose; **(C)** High BMI; **(D)** High alcohol use; **(E)** High processed meat intake; **(F)** High sugar-sweetened beverages intake; **(G)** Physical inactivity; **(H)** Chewing tobacco; **(I)** Kidney dysfunction.

This indicates that the exposure to gout risk factors among Chinese individuals aged 15–39 has rapidly increased over the past 30 years, necessitating focused attention on the future burden of gout in this population and targeted interventions for rapidly growing risk factors. Figure 7 illustrates the temporal trends of exposure to gout risk factors among young adults in China from 1990 to 2021. The detailed exposure values are presented in Supplementary material 6.

## 4 Discussion

The burden of gout among the young population is often overlooked. Existing research tends to focus more on the rapidly increasing absolute burden of gout in the older adult population, while neglecting the similarly rapid rise in burden among the young, despite their lower absolute burden compared to the older adult (3–5). Existing studies have observed a significant increase in the incidence of gout and years lived with disability (YLD) among the young population in high-income North American and East Asian regions, indicating a substantial failure in the prevention and treatment of gout in young patients (6).

From 1990 to 2021, the number of gout prevalent cases among Chinese individuals aged 15–39 increased by 31.9%, compared to a global increase of 66.1%. Meanwhile, the percentage of gout prevalent cases among Chinese individuals aged 15–39, relative to the global 15–39 age group, fell from 35.6% in 1990 to 28.3% in 2021. The proportion of gout patients aged 15–39 in China accounts for the world’s total is decreasing, indicating that the growth rate of gout prevalent cases in other regions is higher than that in China. We retrieved the total percentage change of gout prevalent cases in 204 countries from 1990 to 2021 in GBD 2021. For instance, Qatar had an 868% increase from 1990 to 2021, followed by Maldives (759%) and the United Arab Emirates (678%) over the same period. Compared to these figures, China’s total percentage change in gout prevalent cases over the past 31 years is considerably lower. The prevalent cases in a region is determined by multiplying the prevalence rate by the population size. Our observations reveal that the ASPR for individuals aged 15–39 in China is rising faster than the global average. However, the number of prevalent cases within this age group in China has not increased in tandem, pointing to a population growth rate for this age group that is lower than the global average. We obtained data on the total population of China and the world from 1990 to 2021 from the GBD population. Between 1990 and 2021, the population of individuals aged 15–39 in China decreased from 548,138,948 to 461,444,919, while the global population in the same age group increased from 2,191,797,967 to 2,974,827,509. The proportion of China’s 15–39 age group relative to the world’s decreased significantly, from 25.01% in 1990 to 15.51% in 2021. This can be attributed to the effective reduction in the number of new births in China due to the family planning policy implemented since the 1980s (21), resulting in a relatively stagnant growth rate of the total burden among the young population. The same trend can be observed in the incident cases and DALYs.

Nonetheless, due to China’s large population base, it remains a country with a high absolute burden of gout. China still had the highest number of gout prevalenct cases aged 15–39 in 2021 globally. Additionally, China also experienced the greatest absolute increase in the number of gout prevalent cases in this age group worldwide from 1990 to 2021. The burden of gout among young people in China remains a significant public health issue.

Although the growth of the absolute burden of gout among the young population in China is lower than the global level, China has significantly higher ASPR, ASIR, and ASDR compared to the world average. Moreover, the growth rate of these ASRs in China is also higher than the global average. This is due to the fact that even as the incidence rate rises for each young individual, the overall number of cases does not grow as rapidly due to the decreasing size of the young population. Since age-standardized rates are not affected by population structure, future research should pay more attention to changes in these rates. This approach would more accurately capture the rise in individual risk of disease that may be obscured by absolute numbers.

The higher ASRs in the young population of China indicates that the intensity of gout risk exposure and disease burden among young people in China is significantly higher than the global average. This can be attributed to the rapid westernization of the Chinese diet, leading to high purine and sugary beverage intake, as well as the younger trend of metabolic syndrome. It also reflects the improvement of medical diagnostic capabilities in China and the insufficient management of gout complications.

The significant gender disparities observed in this study are consistent with previous research, with males experiencing a higher burden of gout across all metrics (9). These disparities are likely influenced by a combination of biological factors, including testosterone levels and xanthine oxidase activity (22), as well as behavioral factors such as alcohol intake and dietary preferences. Although the disease burden of males and females both increases with age, the growth rates show significant gender differences. The analysis for each age subgroup by sex for 2021 (Figure 3) reveals that, during the 15–19 to 20–24 years period, the 5-year total growth rate for males was 1,053% (from 6.57 [95% UI: 2.27–13.99] to 75.82 [95% UI: 47.06–112.32]), while for females it was 690% (from 2.96 [95% UI: 0.67–6.22] to 23.39 [95% UI: 10.32–40.1]). But during the 30–34 to 35–39 years stage, the growth rate for females slightly exceeded that of males, with 67.1% (from 150.38 [95% UI: 85.16–233.37] to 251.34 [95% UI: 156.3–374.97]) compared to 66.9% (from 655.46 [95% UI: 406.4–949.15] to 1,094.44 [95% UI: 719.18–1,557.69]). Similar patterns were observed in incidence and DALY rates.

The surge in gout burden among males during early adulthood is attributed to the increase in testosterone levels during adolescence, which significantly enhances the activity of the urate transporter 1 (URAT1) gene promoter (22). Conversely, the delayed onset of gout acceleration in females, typically observed around the ages of 35–39, can be attributed to the role of estrogen in enhancing the clearance of uric acid by the kidneys, which is facilitated by its ability to regulate renal tubular excretion through ABCG2 (23, 24). However, this clearance effect gradually decreases as estrogen levels decline. Gender-divergent behavioral patterns, particularly the elevated alcohol consumption and preference for purine-rich diets among young males, contribute to the early onset of hyperuricemia in males. In contrast, the midlife acceleration in females coincides with sociocultural shifts, such as reduced physical activity and increased intake of sugar-sweetened beverages, which amplify gout risks with declining estrogen levels. These results suggest that the rapid-growth phase of gout burden occurs 5–10 years earlier in males than in females, highlighting the need for early preventive interventions targeting young males.

The joinpoint regression analysis has pointed a notable acceleration in trends post-2019. Changes in dietary habits and lifestyle during the pandemic may affect uric acid levels. However, there is no data on dietary habits during the pandemic in China. A study in Italy showed a significant increase in the intake of sweet or salty foods during the lockdown (25). Another review also indicated that lockdowns may increase the intake of processed meats and decrease the intake of fruits and vegetables (26). Therefore, the rising burden of COVID-19 in China since 2019 may be related to changes in dietary structure caused by COVID-19 control policies. Along with this, COVID-19 containment strategies led to a remarkable increase in physical inactivity among urban youth, with the prevalence of inactive students rising extensively from 21.3 to 65.6% (27), establishing a “double-hit” scenario where overconsumption of purines combined with increased metabolic risks. This upward trend since 2019 needs to be supported by more data, awaiting confirmation by subsequent updated GBD data.

ARIMA model shows that females demonstrate a predictable rise in ASPR, ASIR and ASDR. The ASIR is rising faster than the ASPR, which means that new cases have a greater driving force on the epidemic than long-term survival with the disease. The 6% ASDR increase predicts women with gout are experiencing more severe symptoms, prolonged disease duration, and suboptimal treatment effectiveness. Despite males facing elevated baseline burdens and accelerating ASDR growth, their ASPR and ASIR exhibit downward trends. In our sensitivity analysis, by adjusting the (*p*, *d*, *q*) parameters and re-analyzing the data after removing the most recent 3 years, we found that the gradual decline in male ASPR and ASIR, as well as the continuous increase in female rates, remained unchanged, reinforcing the certainty of this gender difference.

In the next 15 years, both the ASPR and ASIR of gout among Chinese males aged 15–39 show a downward trend, while the ASDR shows an upward trend. The stabilization and potential decline in ASPR and ASIR among males in the future may be related to the already high baseline burden of the disease. The elevated baseline levels suggest limited room for further increases. Additionally, this downward trend also reflects, to some extent, the effectiveness of screening and treatment efforts within the healthcare system for gout. The rising ASDR indicates that although the incidence and prevalence of gout in the study population are decreasing, the condition of those already affected by gout is worsening due to certain reasons. This trend may be attributable to the cumulative effect of gout complications, which results in a marked increase in Years Lived with Disability (YLD) within the DALYs. The rising presence of comorbidities such as obesity, hypertension, and diabetes in male gout patients likely exacerbates the overall health burden imposed by gout. This is further supported by the SEV analysis, which indicates a progressive elevation in metabolic risk factors within this demographic cohort.

The increasing trend of gout in Chinese women aged 15–39 may be attributed to changes in their social status and lifestyle. As the educational level of Chinese women aged 15–39 continues to rise, a greater proportion are taking on office jobs (28), which typically involve less physical exertion. This shift in occupation, coupled with improved economic status, has led to changes in consumption habits among urban women, such as increased consumption of high-sugar milk tea and fast food, both of which are associated with a higher risk of developing gout (29–31).

Therefore, it is necessary to place greater emphasis on gout-related health education, prevention, and disease control specifically targeting females. Targeted screening for hyperuricemia and joint X-rays, coupled with educational interventions and lifestyle modifications, should be strengthened among young Chinese women to address unhealthy dietary habits and lifestyle choices. On the other hand, efforts can be focused on the promotion of social sports initiatives. While promoting traditional competitive sports that are more popular among men, more attention should be paid to types of exercise that women tend to favor, such as yoga and aerobics. Additionally, in response to the increasing consumption of high-sugar beverages among women, pilot programs such as taxing beverage companies could be gradually implemented. At the same time, alternative sweetening options with lower metabolic risks, such as sugar substitutes, could be introduced. Meanwhile, for males, management of gout complications should be reinforced, starting with the reduction of metabolic risks. Efforts should focus on promoting weight management, blood glucose control, and blood pressure control to reduce the annually increasing ASDR.

From the sensitivity analysis, we can see that the predictive results of the ARIMA model largely depend on the selected (*p*, *d*, *q*) parameters and are sensitive to data changes and missing values. After removing the data from 2019 to 2021 related to the pandemic, we conducted the forecasts again. Some indicators exhibited fluctuations in the data, while others, such as ASIR of Both and ASDR of Both, showed differences in trends. Therefore, it is undeniable that the recent data fluctuations have an impact on the predictive performance. Whether the trend differences caused by the short-term fluctuations in the data since 2019 will persist in the future will depend on future GBD data for confirmation. More comprehensive and accurate data will enhance the precision of the modeling.

Future research can incorporate multiple forecasting models for a combined analysis. ARIMA model is more suitable for short-term, univariate simple forecasts, with the advantage of being transparent and easy to interpret. The BAPC (Bayesian Age-Period-Cohort) model and machine learning predictions can serve as complements to the ARIMA model. BAPC excels at decomposing age, period, and cohort effects and is particularly suitable for analyzing the long-term trends of disease incidence, capable of integrating prior knowledge through Bayesian methods (32). Machine learning (such as XGBoost, LSTM) can automatically capture complex nonlinear relationships, is compatible with multi-source data, and provides more accurate predictions in big data scenarios (33, 34).

The rising exposure to gout risk factors indicates that, although the growth rate of gout in young adults is relatively moderate compared to the older adult (6), their dietary and metabolic risks are rapidly increasing. It is necessary to strengthen uric acid screening for young adults and intervene during the asymptomatic hyperuricemia stage of gout (35). Based on the SEV data, controlling high-risk dietary intake and reducing metabolic risks through controlling high BMI are crucial for reducing the incidence of gout in this population.

This study is not without its limitations. The findings are influenced by the constraints inherent in the GBD dataset, such as potential estimation biases and the dependency on modeled data. The GBD data are not derived from direct, real-world surveys, but rather are estimated from various data sources through modeling (12). This reliance on modeled data introduces the potential for estimation biases particularly in low- and middle-income countries, where primary epidemiological data on gout prevalence and disability remain scarce.

Heterogeneity in diagnostic criteria across studies poses a significant challenge to data consistency. The GBD analysis adopts physician-diagnosed gout based on the ACR criteria as the reference standard, yet many included studies utilize alternative definitions, such as self-reported diagnoses or administrative coding. In low- and middle-income countries, where access to rheumatological expertise and serum urate testing is limited, misclassification between gout and clinically similar conditions like pseudogout may inflate prevalence estimates. Therefore, the results should be interpreted with caution, acknowledging that the real-world scenario might be more complex than what is captured by the model.

The data provided by GBD does not fully reflect the detailed domestic burden within China. 1.41 billion Chinese people are spread across 34 regions, with diverse ethnicities, socioeconomic levels, behavioral contexts, and dietary differences, all of which contribute to significant heterogeneity in China’s gout burden. Some studies have reported geographical and population differences in hyperuricemia in China (35, 36), but there is still a lack of domestic reports specifically on gout, especially data from the post-COVID-19 era. In the future, we will focus on the disparities in the distribution of gout within China and conduct more research at the domestic level. Meanwhile, domestic databases in China, such as the China Health and Nutrition Survey (CHNS) and the National Population Health Data Center (NPHDC) (37, 38), offer more detailed domestic data. Joint analyses of multiple databases can address this deficiency, and we look forward to seeing this aspect in future research.

From the GBD 2021 dataset, we extracted the SEV data for 73 available risk factors among the 15–39 age group in China and selected 9 risk factors that are closely related to gout. Among them, hypertension, high fasting glucose, high BMI, and kidney dysfunction reflect the metabolic risks of the study population. High alcohol use, high processed meat intake, and high sugar-sweetened beverage intake reflect the dietary risks of the study population. Physical inactivity and chewing tobacco reflect the behavioral risks of the study population. However, these 9 risk factors do not fully capture all the risk factors for gout, such as the use of diuretics, high seafood intake, strenuous exercise, or stress. Additionally, high red meat intake was not included in our study due to the lack of confidence intervals. We look forward to more comprehensive data in future SEV releases.

SEV treats these risk factors as independent of each other. However, this approach has limitations in practice. There are significant pathophysiological synergistic effects among metabolic risk factors. There is a mutually promoting pathophysiological relationship between high blood glucose and high blood pressure (39–41). Obesity will increase the burden on the cardiovascular system, thereby increasing the risk of hypertension (42). Individuals with a genetically high BMI exhibit an increased risk of impaired kidney function (43). On the other hand, some of these risk factors are also outcomes of gout. Kidney dysfunction serves as both a risk factor for and a consequence of gout (44–46). Hyperuricemia and gout are associated with an increased risk of diabetes (47–49). This reverse causality can make it difficult to discern whether the increase in exposure to risk factors leads to a rise in the burden of gout, or whether the growing burden of gout results in an increase in the population’s exposure to risk factors.

We presented the SEVs of risk factors closely related to gout in the study population, but we did not further analyze the contribution of various exposure factors to the burden of gout. This could be achieved by collecting various literature to comprehensively calculate the relative risk (RR) of each risk factor for gout outcomes, thereby calculating the population attributable fraction (PAF). Some GBD studies have adopted this approach, but the workload is substantial (50). In GBD 2021, gout data were only provided for two risk factors (high BMI and kidney dysfunction), which we believe does not fully reflect the overall picture of attributable gout burden. Therefore, we look forward to more comprehensive risk attribution data in future research. This is crucial for the development of gout prevention policies.

## 5 Conclusion

The findings of this study highlight a concerning trend of early-onset gout in China among the young adults. The significant rise in the prevalence and incidence of gout among young adults necessitates a reevaluation of current public health strategies, which have traditionally focused on older populations. The study underscores the urgency of gender- and age-specific interventions to address the dietary and lifestyle factors contributing to the increased burden of gout in young individuals.

## Data Availability

The original contributions presented in the study are included in the article/Supplementary material, further inquiries can be directed to the corresponding author.
