# Supplementary material for: Systematic analysis of gout burden among young adults in China from 1990 to 2021: findings from the global burden of disease study 2021
Source: Front Public Health. 2025 Jun 9;13:1613801. doi: 10.3389/fpubh.2025.1613801 (PMC12183959; doi:10.3389/fpubh.2025.1613801)
Supplement: Supplementary file 1 [file Data_Sheet_1.pdf]

Supplementary Material 1

The calculated World Standard Population ratios

Age name	Calculated Ratios
<5 years	8.34041594858421
5-9 years	8.70640995908253
10-14 years	8.44767598582997
15-19 years	7.90713389113232
20-24 years	7.56724626687674
25-29 years	7.45554274666252
30-34 years	7.66003183153991
35-39 years	7.10735008990193
40-44 years	6.33922695564581
45-49 years	6.00029688011654
50-54 years	5.63810750685125
55-59 years	5.01470392853073
60-64 years	4.05567766536139
65-69 years	3.49549878214835
70-74 years	2.60841471090543
75-79 years	1.67125205974324
80-84 years	1.10986105400057
85-89 years	0.579391003156452
90-94 years	0.226695889914593
95+ years	0.0690668440155

Supplementary Material 2

The ranking of EAPC in crude incidence rate across 204 countries and regions from 1990-2021

Rank	location	PVALUE	EAPC(95%CI)
1	Republic of Maldives	0	3 (2.51,3.5)
2	United Arab Emirates	0	2.09 (1.73,2.44)
3	Kingdom of Saudi Arabia	0	1.83 (1.72,1.93)
4	Islamic Republic of Iran	0	1.83 (1.57,2.1)
5	United States of America	0	1.8 (1.69,1.91)
6	State of Libya	0	1.8 (1.64,1.96)
7	Islamic Republic of Afghanistan	0	1.79 (1.18,2.4)
8	Republic of Botswana	0	1.78 (1.65,1.9)
9	People's Democratic Republic of Algeria	0	1.67 (1.53,1.81)
10	Hashemite Kingdom of Jordan	0	1.57 (1.5,1.64)
11	Republic of Cabo Verde	0	1.57 (1.41,1.74)
12	Republic of Singapore	0	1.55 (1.43,1.68)
13	Sultanate of Oman	0	1.55 (1.14,1.96)
14	Saint Lucia	0	1.54 (1.46,1.63)
15	Republic of Nicaragua	0	1.52 (1.46,1.58)
16	Republic of Seychelles	0	1.47 (1.42,1.52)
17	Republic of Peru	0	1.46 (1.44,1.47)
18	Czech Republic	0	1.44 (1.16,1.73)
19	Saint Vincent and the Grenadines	0	1.43 (1.32,1.54)
20	Republic of Trinidad and Tobago	0	1.43 (1.25,1.61)
21	Dominican Republic	0	1.42 (1.37,1.46)
22	Socialist Republic of Viet Nam	0	1.41 (1.28,1.53)

23	Republic of Equatorial Guinea	0	1.4 (1.24,1.57)
24	Republic of Tunisia	0	1.39 (1.28,1.51)
25	Kingdom of Thailand	0	1.36 (1.24,1.48)
26	Democratic Republic of Sao Tome and Principe	0	1.36 (1.26,1.47)
27	Lebanese Republic	0	1.35 (1.21,1.49)
28	People's Republic of China	0	1.34 (1.19,1.49)
29	Federative Republic of Brazil	0	1.34 (1.3,1.38)
30	Kingdom of Spain	0	1.34 (1.11,1.58)
31	Republic of Ecuador	0	1.31 (1.28,1.35)
32	Mongolia	0	1.29 (1.19,1.4)
33	Romania	0	1.29 (1.12,1.46)
34	Plurinational State of Bolivia	0	1.29 (1.23,1.36)
35	Republic of Indonesia	0	1.28 (1.24,1.33)
36	Republic of Panama	0	1.24 (1.16,1.33)
37	Taiwan (Province of China)	0	1.23 (0.65,1.8)
38	Republic of Turkey	0	1.23 (1.17,1.29)
39	Kingdom of Bhutan	0	1.23 (1.14,1.32)
40	Australia	0	1.22 (1.12,1.32)
41	Portuguese Republic	0	1.22 (0.98,1.45)
42	Belize	0	1.22 (1.13,1.31)
43	Bolivarian Republic of Venezuela	0	1.21 (1.17,1.26)
44	Hellenic Republic	0	1.2 (0.78,1.62)
45	Kingdom of Morocco	0	1.16 (1.07,1.25)
46	Republic of El Salvador	0	1.16 (1.06,1.25)
47	Saint Kitts and Nevis	0	1.16 (1.08,1.24)

48	Grenada	0	1.14 (1.08,1.19)
49	Republic of Djibouti	0	1.13 (1.08,1.19)
50	Republic of Suriname	0	1.12 (1.01,1.23)
51	Ireland	0	1.12 (1,1.25)
52	Republic of the Marshall Islands	0	1.11 (0.98,1.25)
53	Republic of the Congo	0	1.11 (1.03,1.2)
54	United States Virgin Islands	0	1.11 (1.04,1.17)
55	Kingdom of Cambodia	0	1.1 (0.91,1.3)
56	Republic of Guatemala	0	1.09 (1.01,1.17)
57	Puerto Rico	0	1.09 (1.04,1.14)
58	Jamaica	0	1.08 (0.95,1.21)
59	Republic of Colombia	0	1.06 (1,1.13)
60	Antigua and Barbuda	0	1.05 (0.95,1.16)
61	Republic of Bulgaria	0	1.05 (0.92,1.18)
62	Slovak Republic	0	1.04 (0.85,1.23)
63	Republic of Honduras	0	1.04 (0.99,1.08)
64	Republic of Cuba	0	1.03 (0.76,1.31)
65	Lao People's Democratic Republic	0	1.02 (0.86,1.18)
66	Kingdom of Eswatini	0	1.01 (0.9,1.12)
67	Malaysia	0	1 (0.88,1.12)
68	Turkmenistan	0	1 (0.89,1.11)
69	Republic of Slovenia	0	0.99 (0.89,1.09)
70	Syrian Arab Republic	0	0.99 (0.75,1.23)
71	North Macedonia	0	0.98 (0.88,1.08)
72	Japan	0	0.98 (0.8,1.16)

73	Republic of Korea	0	0.98 (0.89,1.07)
74	State of Qatar	0	0.98 (0.86,1.1)
75	Republic of Estonia	0	0.97 (0.82,1.13)
76	Republic of South Africa	0	0.97 (0.81,1.13)
77	Argentine Republic	0	0.96 (0.89,1.02)
78	Republic of Azerbaijan	0	0.96 (0.7,1.22)
79	Commonwealth of Dominica	0	0.96 (0.76,1.16)
80	Republic of Moldova	0	0.96 (0.64,1.29)
81	Republic of Paraguay	0	0.96 (0.83,1.09)
82	Republic of the Union of Myanmar	0	0.95 (0.91,0.98)
83	Kingdom of Bahrain	0	0.95 (0.73,1.18)
84	Arab Republic of Egypt	0	0.95 (0.81,1.09)
85	Republic of Armenia	0	0.93 (0.55,1.31)
86	Hungary	0	0.93 (0.74,1.12)
87	Republic of Haiti	0	0.93 (0.78,1.07)
88	Republic of Costa Rica	0	0.93 (0.83,1.03)
89	Republic of Yemen	0	0.93 (0.74,1.13)
90	Tokelau	0	0.93 (0.84,1.03)
91	Kingdom of Lesotho	0	0.92 (0.78,1.06)
92	Solomon Islands	0	0.9 (0.8,1.01)
93	Republic of Iraq	0	0.9 (0.77,1.02)
94	United Mexican States	0	0.9 (0.8,1.01)
95	Union of the Comoros	0	0.9 (0.83,0.98)
96	Democratic Socialist Republic of Sri Lanka	0	0.89 (0.83,0.95)
97	Republic of Palau	0	0.88 (0.63,1.13)

98	Republic of Poland	0	0.87 (0.57,1.17)
99	Republic of Uzbekistan	0	0.87 (0.67,1.07)
100	Republic of the Philippines	0	0.86 (0.81,0.92)
101	Republic of Cyprus	0	0.84 (0.67,1.02)
102	Republic of Chile	0	0.84 (0.68,1.01)
103	Republic of Namibia	0	0.84 (0.79,0.89)
104	Republic of Mauritius	0	0.83 (0.78,0.88)
105	Republic of Serbia	0	0.82 (0.68,0.96)
106	Republic of Fiji	0	0.81 (0.76,0.87)
107	Commonwealth of the Bahamas	0	0.8 (0.65,0.94)
108	Kingdom of Tonga	0	0.78 (0.61,0.94)
109	State of Israel	0	0.78 (0.69,0.86)
110	Republic of Italy	0	0.77 (0.58,0.97)
111	Republic of Kenya	0	0.77 (0.69,0.84)
112	Republic of Sudan	0	0.77 (0.75,0.79)
113	Republic of Latvia	0	0.76 (0.57,0.94)
114	State of Kuwait	0	0.76 (0.71,0.81)
115	Republic of Ghana	0	0.76 (0.72,0.8)
116	Republic of Niue	0	0.76 (0.65,0.88)
117	Brunei Darussalam	0	0.75 (0.64,0.86)
118	State of Eritrea	0	0.75 (0.71,0.79)
119	Republic of Zambia	0	0.75 (0.73,0.77)
120	Bermuda	0	0.75 (0.67,0.83)
121	Democratic People's Republic of Korea	0	0.74 (0.59,0.9)
122	Barbados	0	0.74 (0.65,0.83)

123	Eastern Republic of Uruguay	0	0.74 (0.71,0.78)
124	Republic of Guyana	0	0.73 (0.55,0.91)
125	Republic of Malta	0	0.72 (0.57,0.87)
126	Republic of Belarus	0	0.72 (0.44,0.99)
127	Ukraine	0	0.72 (0.45,0.98)
128	Russian Federation	0	0.72 (0.41,1.04)
129	Republic of Kazakhstan	0	0.71 (0.47,0.95)
130	Bosnia and Herzegovina	0	0.69 (0.56,0.82)
131	Republic of Liberia	0	0.67 (0.37,0.96)
132	Republic of Croatia	0	0.66 (0.55,0.77)
133	Republic of Zimbabwe	0	0.66 (0.57,0.75)
134	Burkina Faso	0	0.63 (0.58,0.67)
135	Republic of Cote d'Ivoire	0	0.63 (0.56,0.71)
136	Togolese Republic	0	0.63 (0.58,0.68)
137	People's Republic of Bangladesh	0	0.62 (0.58,0.65)
138	Montenegro	0	0.61 (0.47,0.75)
139	Islamic Republic of Pakistan	0	0.61 (0.59,0.64)
140	Republic of Cameroon	0	0.6 (0.51,0.68)
141	Georgia	0	0.59 (0.39,0.79)
142	Palestine	0	0.56 (0.44,0.67)
143	Federated States of Micronesia	0	0.55 (0.48,0.62)
144	United Republic of Tanzania	0	0.55 (0.51,0.59)
145	Canada	0	0.53 (0.42,0.63)
146	Republic of Tajikistan	0	0.53 (0.32,0.75)
147	Gabonese Republic	0	0.51 (0.48,0.54)

148	Republic of San Marino	0.001	0.5 (0.22,0.78)
149	Principality of Andorra	0	0.49 (0.33,0.66)
150	Tuvalu	0	0.49 (0.42,0.56)
151	Republic of Vanuatu	0	0.46 (0.43,0.48)
152	Republic of Iceland	0	0.46 (0.4,0.52)
153	Republic of Senegal	0	0.46 (0.45,0.47)
154	Cook Islands	0	0.45 (0.29,0.62)
155	Independent State of Samoa	0	0.44 (0.26,0.62)
156	Republic of India	0	0.44 (0.38,0.49)
157	French Republic	0	0.41 (0.34,0.49)
158	Republic of Rwanda	0.011	0.41 (0.11,0.71)
159	Republic of Guinea-Bissau	0	0.41 (0.31,0.52)
160	Republic of Austria	0	0.4 (0.21,0.6)
161	Republic of the Gambia	0	0.4 (0.39,0.42)
162	Republic of Benin	0	0.4 (0.38,0.42)
163	Republic of Albania	0.001	0.37 (0.18,0.55)
164	Republic of Malawi	0	0.37 (0.3,0.44)
165	Independent State of Papua New Guinea	0	0.36 (0.31,0.41)
166	New Zealand	0.069	0.35 (-0.01,0.72)
167	Kyrgyz Republic	0.001	0.35 (0.15,0.54)
168	Republic of Lithuania	0	0.35 (0.23,0.47)
169	Federal Republic of Somalia	0.057	0.34 (0,0.69)
170	American Samoa	0	0.34 (0.21,0.48)
171	Islamic Republic of Mauritania	0	0.31 (0.29,0.33)
172	Federal Democratic Republic of Ethiopia	0	0.3 (0.21,0.39)

173	Kingdom of Norway	0	0.29 (0.18,0.4)
174	Grand Duchy of Luxembourg	0	0.29 (0.18,0.39)
175	Republic of Uganda	0	0.29 (0.25,0.32)
176	Swiss Confederation	0	0.28 (0.17,0.38)
177	Federal Democratic Republic of Nepal	0	0.25 (0.21,0.28)
178	Kingdom of Belgium	0	0.24 (0.14,0.34)
179	Republic of Finland	0	0.24 (0.16,0.33)
180	Republic of Mozambique	0	0.24 (0.16,0.32)
181	United Kingdom of Great Britain and Northern Ireland	0.026	0.23 (0.04,0.43)
182	Kingdom of Denmark	0.034	0.23 (0.03,0.44)
183	Republic of South Sudan	0	0.23 (0.14,0.32)
184	Republic of Nauru	0	0.22 (0.14,0.3)
185	Republic of Madagascar	0	0.2 (0.17,0.22)
186	Federal Republic of Germany	0.064	0.18 (0,0.37)
187	Republic of Angola	0	0.18 (0.13,0.23)
188	Republic of Kiribati	0.054	0.17 (0,0.34)
189	Republic of Burundi	0.428	0.09 (-0.13,0.31)
190	Central African Republic	0.002	0.06 (0.03,0.1)
191	Democratic Republic of the Congo	0.061	0.06 (0,0.13)
192	Republic of Chad	0.004	0.05 (0.02,0.08)
193	Republic of Mali	0.327	0.02 (-0.02,0.06)
194	Guam	0.817	0.02 (-0.12,0.15)
195	Republic of Sierra Leone	0.864	-0.01 (-0.09,0.07)
196	Greenland	0.965	-0.01 (-0.34,0.33)
197	Federal Republic of Nigeria	0.579	-0.02 (-0.08,0.05)

198	Republic of Guinea	0.273	-0.04 (-0.12,0.03)
199	Northern Mariana Islands	0.242	-0.2 (-0.52,0.13)
200	Principality of Monaco	0.106	-0.21 (-0.46,0.04)
201	Republic of the Niger	0	-0.28 (-0.39,-0.18)
202	Kingdom of the Netherlands	0.024	-0.35 (-0.64,-0.06)
203	Democratic Republic of Timor-Leste	0	-0.37 (-0.4,-0.33)
204	Kingdom of Sweden	0.003	-0.59 (-0.94,-0.24)

The ranking of EAPC in crude incidence rate across 204 countries and regions from 1990-2021

Rank	location	PVALUE	EAPC(95%CI)
1	Republic of Maldives	0	2.78 (2.33,3.22)
2	United Arab Emirates	0	1.87 (1.6,2.14)
3	Islamic Republic of Iran	0	1.67 (1.45,1.9)
4	Kingdom of Saudi Arabia	0	1.64 (1.55,1.72)
5	United States of America	0	1.64 (1.53,1.74)
6	Republic of Botswana	0	1.61 (1.51,1.71)
7	State of Libya	0	1.58 (1.44,1.73)
8	Islamic Republic of Afghanistan	0	1.52 (1.03,2.01)
9	People's Democratic Republic of Algeria	0	1.51 (1.39,1.62)
10	Sultanate of Oman	0	1.48 (1.12,1.85)
11	Republic of Cabo Verde	0	1.43 (1.3,1.57)
12	Saint Lucia	0	1.41 (1.35,1.47)
13	Republic of Nicaragua	0	1.4 (1.35,1.44)
14	Republic of Equatorial Guinea	0	1.38 (1.24,1.53)

15	Hashemite Kingdom of Jordan	0	1.37 (1.31,1.43)
16	Republic of Trinidad and Tobago	0	1.34 (1.19,1.49)
17	Republic of Peru	0	1.34 (1.33,1.35)
18	Czech Republic	0	1.34 (1.1,1.59)
19	Dominican Republic	0	1.33 (1.3,1.35)
20	Republic of Seychelles	0	1.33 (1.29,1.37)
21	Republic of Singapore	0	1.32 (1.22,1.42)
22	Saint Vincent and the Grenadines	0	1.31 (1.22,1.39)
23	Lebanese Republic	0	1.29 (1.17,1.41)
24	Socialist Republic of Viet Nam	0	1.28 (1.17,1.38)
25	Federative Republic of Brazil	0	1.27 (1.24,1.3)
26	Republic of Ecuador	0	1.26 (1.23,1.29)
27	Republic of Tunisia	0	1.25 (1.15,1.35)
28	Plurinational State of Bolivia	0	1.24 (1.19,1.3)
29	People's Republic of China	0	1.23 (1.14,1.33)
30	Democratic Republic of Sao Tome and Principe	0	1.21 (1.12,1.31)
31	Mongolia	0	1.18 (1.08,1.28)
32	Republic of Indonesia	0	1.18 (1.14,1.22)
33	Romania	0	1.18 (1.04,1.32)
34	Republic of Panama	0	1.16 (1.1,1.23)
35	Kingdom of Thailand	0	1.16 (1.06,1.26)
36	Belize	0	1.14 (1.06,1.22)
37	Grenada	0	1.12 (1.07,1.17)
38	Kingdom of Bhutan	0	1.12 (1.05,1.2)
39	Kingdom of Spain	0	1.11 (0.89,1.33)

40	Republic of Turkey	0	1.11 (1.06,1.15)
41	Bolivarian Republic of Venezuela	0	1.1 (1.07,1.12)
42	Taiwan (Province of China)	0	1.09 (0.6,1.58)
43	Republic of El Salvador	0	1.08 (1,1.16)
44	Saint Kitts and Nevis	0	1.08 (1.02,1.14)
45	Kingdom of Morocco	0	1.07 (0.99,1.15)
46	United States Virgin Islands	0	1.07 (1.02,1.12)
47	Jamaica	0	1.05 (0.96,1.14)
48	Republic of Guatemala	0	1.05 (0.99,1.11)
49	Kingdom of Cambodia	0	1.05 (0.88,1.21)
50	Puerto Rico	0	1.04 (0.99,1.08)
51	Republic of Colombia	0	1.03 (0.98,1.07)
52	Australia	0	1.03 (0.96,1.1)
53	Republic of Suriname	0	1.02 (0.93,1.1)
54	State of Qatar	0	1.02 (0.9,1.13)
55	Republic of Honduras	0	1.01 (0.98,1.04)
56	Portuguese Republic	0	1.01 (0.8,1.22)
57	Republic of Djibouti	0	1.01 (0.96,1.06)
58	Hellenic Republic	0	1.01 (0.6,1.41)
59	Antigua and Barbuda	0	1 (0.92,1.07)
60	Republic of the Marshall Islands	0	1 (0.89,1.11)
61	Slovak Republic	0	0.99 (0.84,1.14)
62	Lao People's Democratic Republic	0	0.98 (0.84,1.12)
63	Republic of Bulgaria	0	0.98 (0.87,1.09)
64	Republic of the Congo	0	0.97 (0.89,1.05)

65	Turkmenistan	0	0.97 (0.87,1.06)
66	Republic of Azerbaijan	0	0.97 (0.74,1.2)
67	Republic of Moldova	0	0.97 (0.69,1.24)
68	Republic of Paraguay	0	0.96 (0.86,1.06)
69	Republic of Cuba	0	0.95 (0.74,1.15)
70	Kingdom of Eswatini	0	0.95 (0.86,1.04)
71	Malaysia	0	0.95 (0.86,1.05)
72	Republic of Armenia	0	0.95 (0.62,1.27)
73	Republic of Estonia	0	0.95 (0.81,1.1)
74	North Macedonia	0	0.94 (0.85,1.03)
75	Ireland	0	0.94 (0.81,1.06)
76	Republic of Slovenia	0	0.94 (0.85,1.03)
77	Commonwealth of Dominica	0	0.92 (0.77,1.06)
78	Kingdom of Bahrain	0	0.92 (0.72,1.13)
79	Kingdom of Lesotho	0	0.92 (0.81,1.03)
80	Republic of South Africa	0	0.92 (0.78,1.06)
81	Republic of Haiti	0	0.91 (0.8,1.03)
82	United Mexican States	0	0.91 (0.86,0.97)
83	Arab Republic of Egypt	0	0.9 (0.79,1.01)
84	Republic of Costa Rica	0	0.9 (0.82,0.98)
85	Republic of Yemen	0	0.88 (0.72,1.05)
86	Argentine Republic	0	0.88 (0.82,0.94)
87	Hungary	0	0.88 (0.73,1.03)
88	Republic of Korea	0	0.86 (0.79,0.93)
89	Republic of Poland	0	0.86 (0.61,1.11)

90	Republic of the Union of Myanmar	0	0.85 (0.82,0.89)
91	Japan	0	0.85 (0.69,1)
92	Republic of Uzbekistan	0	0.84 (0.66,1.02)
93	Republic of the Philippines	0	0.84 (0.79,0.88)
94	Tokelau	0	0.82 (0.74,0.9)
95	Union of the Comoros	0	0.81 (0.75,0.87)
96	Republic of Iraq	0	0.8 (0.7,0.9)
97	Democratic Socialist Republic of Sri Lanka	0	0.79 (0.74,0.84)
98	Republic of Serbia	0	0.79 (0.66,0.91)
99	Republic of Chile	0	0.78 (0.65,0.91)
100	Republic of Palau	0	0.78 (0.56,0.99)
101	Republic of Namibia	0	0.77 (0.72,0.82)
102	Republic of Mauritius	0	0.76 (0.73,0.79)
103	Syrian Arab Republic	0	0.76 (0.53,0.99)
104	Solomon Islands	0	0.76 (0.67,0.85)
105	Commonwealth of the Bahamas	0	0.75 (0.63,0.86)
106	Republic of Cyprus	0	0.75 (0.6,0.9)
107	Republic of Latvia	0	0.74 (0.58,0.9)
108	Barbados	0	0.74 (0.66,0.81)
109	Republic of Sudan	0	0.74 (0.72,0.76)
110	Republic of Guyana	0	0.73 (0.58,0.87)
111	Republic of Ghana	0	0.73 (0.7,0.77)
112	Republic of Fiji	0	0.73 (0.69,0.77)
113	Russian Federation	0	0.73 (0.45,1)
114	Republic of Kazakhstan	0	0.72 (0.5,0.93)

115	Republic of Belarus	0	0.71 (0.47,0.95)
116	Ukraine	0	0.71 (0.48,0.94)
117	Bermuda	0	0.7 (0.65,0.76)
118	Brunei Darussalam	0	0.69 (0.58,0.8)
119	State of Eritrea	0	0.69 (0.66,0.72)
120	Republic of Kenya	0	0.69 (0.63,0.76)
121	Republic of Liberia	0	0.68 (0.44,0.92)
122	Republic of Zambia	0	0.68 (0.66,0.69)
123	Bosnia and Herzegovina	0	0.68 (0.58,0.79)
124	Eastern Republic of Uruguay	0	0.67 (0.63,0.71)
125	State of Israel	0	0.66 (0.58,0.74)
126	Republic of Niue	0	0.66 (0.56,0.76)
127	Democratic People's Republic of Korea	0	0.66 (0.56,0.76)
128	Republic of Malta	0	0.65 (0.53,0.78)
129	State of Kuwait	0	0.65 (0.59,0.7)
130	Republic of Croatia	0	0.65 (0.56,0.75)
131	Kingdom of Tonga	0	0.64 (0.49,0.78)
132	Republic of Italy	0	0.64 (0.48,0.81)
133	Georgia	0	0.61 (0.43,0.79)
134	Montenegro	0	0.6 (0.47,0.72)
135	Burkina Faso	0	0.59 (0.55,0.63)
136	Islamic Republic of Pakistan	0	0.59 (0.57,0.61)
137	Republic of Cameroon	0	0.57 (0.5,0.64)
138	Togolese Republic	0	0.57 (0.53,0.61)
139	Republic of Zimbabwe	0	0.57 (0.51,0.64)

140	People's Republic of Bangladesh	0	0.56 (0.52,0.59)
141	Republic of Cote d'Ivoire	0	0.56 (0.5,0.62)
142	Republic of Tajikistan	0	0.56 (0.37,0.75)
143	Palestine	0	0.53 (0.43,0.62)
144	Federated States of Micronesia	0	0.53 (0.47,0.6)
145	Tuvalu	0	0.52 (0.47,0.58)
146	United Republic of Tanzania	0	0.5 (0.47,0.54)
147	Canada	0	0.48 (0.38,0.58)
148	Gabonese Republic	0	0.47 (0.45,0.49)
149	Republic of Senegal	0	0.44 (0.43,0.45)
150	Republic of Rwanda	0.003	0.43 (0.17,0.68)
151	Republic of Vanuatu	0	0.43 (0.41,0.44)
152	Republic of Albania	0	0.42 (0.25,0.59)
153	Republic of Benin	0	0.42 (0.4,0.44)
154	Republic of India	0	0.41 (0.36,0.47)
155	Republic of Iceland	0	0.4 (0.34,0.45)
156	Republic of Guinea-Bissau	0	0.4 (0.32,0.49)
157	Kyrgyz Republic	0	0.4 (0.23,0.57)
158	Republic of the Gambia	0	0.38 (0.37,0.39)
159	Republic of San Marino	0.004	0.37 (0.14,0.6)
160	Republic of Lithuania	0	0.37 (0.27,0.47)
161	Cook Islands	0	0.36 (0.22,0.49)
162	Independent State of Samoa	0	0.36 (0.21,0.5)
163	Federal Republic of Somalia	0.015	0.35 (0.08,0.63)
164	French Republic	0	0.34 (0.27,0.42)

165	Republic of Austria	0	0.34 (0.19,0.49)
166	Independent State of Papua New Guinea	0	0.33 (0.28,0.38)
167	Republic of Malawi	0	0.32 (0.26,0.39)
168	Federal Democratic Republic of Ethiopia	0	0.32 (0.24,0.4)
169	Islamic Republic of Mauritania	0	0.32 (0.3,0.34)
170	Principality of Andorra	0	0.32 (0.18,0.46)
171	New Zealand	0.121	0.3 (-0.07,0.67)
172	Republic of Mozambique	0	0.28 (0.2,0.35)
173	Republic of Uganda	0	0.28 (0.25,0.32)
174	Federal Democratic Republic of Nepal	0	0.24 (0.2,0.28)
175	American Samoa	0	0.23 (0.12,0.35)
176	Kingdom of Norway	0.001	0.22 (0.1,0.34)
177	Grand Duchy of Luxembourg	0	0.22 (0.14,0.3)
178	Republic of Nauru	0	0.22 (0.16,0.29)
179	Republic of Finland	0	0.22 (0.15,0.29)
180	United Kingdom of Great Britain and Northern Ireland	0.011	0.21 (0.06,0.36)
181	Swiss Confederation	0	0.21 (0.12,0.29)
182	Republic of Madagascar	0	0.2 (0.19,0.22)
183	Republic of Kiribati	0.01	0.19 (0.05,0.32)
184	Kingdom of Belgium	0	0.19 (0.11,0.27)
185	Kingdom of Denmark	0.044	0.18 (0.01,0.34)
186	Republic of Angola	0	0.18 (0.13,0.23)
187	Republic of South Sudan	0.001	0.16 (0.08,0.24)
188	Republic of Burundi	0.149	0.15 (-0.05,0.34)
189	Federal Republic of Germany	0.056	0.15 (0,0.29)

190	Democratic Republic of the Congo	0.005	0.09 (0.03,0.14)
191	Republic of Chad	0	0.08 (0.06,0.11)
192	Republic of Mali	0.001	0.08 (0.04,0.12)
193	Republic of Sierra Leone	0.076	0.06 (0,0.13)
194	Central African Republic	0.002	0.05 (0.02,0.08)
195	Guam	0.753	0.02 (-0.09,0.12)
196	Republic of Guinea	0.626	0.02 (-0.05,0.09)
197	Federal Republic of Nigeria	0.829	-0.01 (-0.07,0.05)
198	Greenland	0.836	-0.03 (-0.28,0.23)
199	Republic of the Niger	0	-0.19 (-0.27,-0.11)
200	Principality of Monaco	0.035	-0.23 (-0.43,-0.03)
201	Northern Mariana Islands	0.072	-0.25 (-0.52,0.01)
202	Democratic Republic of Timor-Leste	0	-0.28 (-0.31,-0.25)
203	Kingdom of the Netherlands	0.014	-0.31 (-0.54,-0.08)
204	Kingdom of Sweden	0.001	-0.64 (-0.97,-0.3)

The ranking of EAPC in crude incidence rate across 204 countries and regions from 1990-2021

Rank	location	PVALUE	EAPC(95%CI)
1	Republic of Maldives	0	2.99 (2.5,3.48)
2	United Arab Emirates	0	2.07 (1.71,2.42)
3	Islamic Republic of Iran	0	1.83 (1.57,2.09)
4	Kingdom of Saudi Arabia	0	1.8 (1.7,1.9)
5	Islamic Republic of Afghanistan	0	1.8 (1.2,2.4)
6	United States of America	0	1.77 (1.66,1.89)
7	Republic of Botswana	0	1.77 (1.64,1.91)
8	State of Libya	0	1.76 (1.6,1.92)
9	People's Democratic Republic of Algeria	0	1.66 (1.52,1.8)
10	Republic of Cabo Verde	0	1.6 (1.44,1.77)
11	Sultanate of Oman	0	1.58 (1.17,1.99)
12	Hashemite Kingdom of Jordan	0	1.57 (1.5,1.64)
13	Republic of Singapore	0	1.56 (1.44,1.68)
14	Saint Lucia	0	1.51 (1.42,1.6)
15	Republic of Nicaragua	0	1.5 (1.44,1.56)
16	Republic of Seychelles	0	1.45 (1.39,1.51)
17	Republic of Equatorial Guinea	0	1.43 (1.27,1.6)
18	Czech Republic	0	1.42 (1.14,1.69)
19	Republic of Peru	0	1.42 (1.39,1.45)
20	Republic of Trinidad and Tobago	0	1.42 (1.25,1.59)
21	Saint Vincent and the Grenadines	0	1.41 (1.3,1.51)
22	Socialist Republic of Viet Nam	0	1.39 (1.27,1.52)
23	Lebanese Republic	0	1.37 (1.23,1.52)

24	Kingdom of Spain	0	1.37 (1.13,1.6)
25	Democratic Republic of Sao Tome and Principe	0	1.37 (1.28,1.47)
26	Dominican Republic	0	1.36 (1.32,1.41)
27	People's Republic of China	0	1.35 (1.2,1.49)
28	Kingdom of Thailand	0	1.34 (1.22,1.45)
29	Federative Republic of Brazil	0	1.33 (1.28,1.37)
30	Republic of Tunisia	0	1.33 (1.22,1.44)
31	Mongolia	0	1.29 (1.19,1.39)
32	Plurinational State of Bolivia	0	1.28 (1.2,1.35)
33	Republic of Ecuador	0	1.28 (1.24,1.33)
34	Romania	0	1.27 (1.1,1.44)
35	Kingdom of Bhutan	0	1.26 (1.17,1.34)
36	Republic of Indonesia	0	1.25 (1.21,1.3)
37	Republic of Turkey	0	1.24 (1.18,1.31)
38	Portuguese Republic	0	1.23 (1,1.46)
39	Australia	0	1.23 (1.12,1.33)
40	Taiwan (Province of China)	0	1.2 (0.63,1.77)
41	Belize	0	1.18 (1.08,1.27)
42	Bolivarian Republic of Venezuela	0	1.18 (1.14,1.23)
43	Republic of Panama	0	1.17 (1.09,1.26)
44	Saint Kitts and Nevis	0	1.17 (1.09,1.25)
45	Kingdom of Morocco	0	1.16 (1.07,1.25)
46	Hellenic Republic	0	1.16 (0.75,1.58)
47	Republic of El Salvador	0	1.15 (1.07,1.23)
48	Grenada	0	1.14 (1.08,1.19)

49	Republic of Djibouti	0	1.14 (1.07,1.2)
50	Kingdom of Cambodia	0	1.13 (0.94,1.32)
51	Republic of the Congo	0	1.11 (1.02,1.2)
52	Ireland	0	1.11 (0.99,1.24)
53	United States Virgin Islands	0	1.1 (1.03,1.16)
54	Puerto Rico	0	1.09 (1.03,1.14)
55	Republic of Guatemala	0	1.08 (1,1.15)
56	Republic of the Marshall Islands	0	1.08 (0.95,1.22)
57	Republic of Suriname	0	1.07 (0.96,1.18)
58	Republic of Colombia	0	1.05 (0.99,1.12)
59	Jamaica	0	1.04 (0.92,1.16)
60	Antigua and Barbuda	0	1.04 (0.94,1.13)
61	Republic of Bulgaria	0	1.02 (0.89,1.16)
62	Republic of Cuba	0	1.02 (0.75,1.29)
63	Slovak Republic	0	1.01 (0.83,1.2)
64	Lao People's Democratic Republic	0	1.01 (0.84,1.18)
65	Malaysia	0	1.01 (0.89,1.12)
66	Republic of Moldova	0	1.01 (0.68,1.34)
67	Republic of Korea	0	1 (0.91,1.08)
68	Turkmenistan	0	0.99 (0.88,1.1)
69	Republic of Estonia	0	0.99 (0.85,1.14)
70	Republic of Honduras	0	0.99 (0.94,1.04)
71	Arab Republic of Egypt	0	0.99 (0.85,1.13)
72	Japan	0	0.98 (0.8,1.16)
73	North Macedonia	0	0.98 (0.88,1.08)

74	Kingdom of Bahrain	0	0.97 (0.74,1.19)
75	State of Qatar	0	0.96 (0.85,1.08)
76	Kingdom of Eswatini	0	0.96 (0.84,1.08)
77	Syrian Arab Republic	0	0.96 (0.71,1.2)
78	Republic of Haiti	0	0.95 (0.8,1.09)
79	Republic of South Africa	0	0.95 (0.79,1.12)
80	Republic of Iraq	0	0.95 (0.81,1.1)
81	Republic of Armenia	0	0.94 (0.56,1.32)
82	Republic of Slovenia	0	0.94 (0.83,1.04)
83	Republic of the Union of Myanmar	0	0.93 (0.88,0.97)
84	Republic of Azerbaijan	0	0.93 (0.67,1.19)
85	Republic of Paraguay	0	0.93 (0.8,1.07)
86	Republic of Costa Rica	0	0.92 (0.82,1.01)
87	Republic of Yemen	0	0.92 (0.72,1.12)
88	Union of the Comoros	0	0.92 (0.84,1)
89	Commonwealth of Dominica	0	0.92 (0.72,1.12)
90	Tokelau	0	0.92 (0.81,1.03)
91	Argentine Republic	0	0.92 (0.85,1)
92	Solomon Islands	0	0.9 (0.8,1.01)
93	Democratic Socialist Republic of Sri Lanka	0	0.9 (0.81,0.98)
94	Republic of Poland	0	0.89 (0.59,1.18)
95	Hungary	0	0.89 (0.7,1.07)
96	Republic of Uzbekistan	0	0.88 (0.67,1.09)
97	United Mexican States	0	0.88 (0.77,0.98)
98	Republic of Palau	0	0.88 (0.65,1.12)

99	Kingdom of Lesotho	0	0.86 (0.71,1.01)
100	Republic of the Philippines	0	0.85 (0.8,0.9)
101	Republic of Cyprus	0	0.84 (0.66,1.02)
102	Republic of Namibia	0	0.83 (0.77,0.9)
103	Republic of Fiji	0	0.82 (0.76,0.88)
104	Republic of Mauritius	0	0.81 (0.76,0.87)
105	Republic of Chile	0	0.81 (0.65,0.98)
106	Republic of Sudan	0	0.81 (0.78,0.83)
107	Republic of Serbia	0	0.79 (0.65,0.93)
108	Republic of Kenya	0	0.79 (0.71,0.87)
109	Republic of Latvia	0	0.78 (0.6,0.95)
110	Republic of Italy	0	0.78 (0.58,0.97)
111	Republic of Ghana	0	0.78 (0.73,0.83)
112	Brunei Darussalam	0	0.78 (0.67,0.9)
113	Commonwealth of the Bahamas	0	0.78 (0.64,0.93)
114	Kingdom of Tonga	0	0.77 (0.6,0.94)
115	State of Eritrea	0	0.77 (0.72,0.83)
116	Republic of Zambia	0	0.76 (0.72,0.79)
117	Eastern Republic of Uruguay	0	0.75 (0.69,0.8)
118	Republic of Niue	0	0.75 (0.63,0.86)
119	State of Israel	0	0.75 (0.67,0.84)
120	Russian Federation	0	0.74 (0.42,1.06)
121	Democratic People's Republic of Korea	0	0.74 (0.59,0.89)
122	State of Kuwait	0	0.74 (0.69,0.8)
123	Republic of Kazakhstan	0	0.73 (0.5,0.97)

124	Republic of Belarus	0	0.73 (0.46,0.99)
125	Ukraine	0	0.72 (0.46,0.98)
126	Bermuda	0	0.72 (0.64,0.8)
127	Republic of Malta	0	0.72 (0.58,0.87)
128	Republic of Guyana	0	0.71 (0.53,0.89)
129	Barbados	0	0.7 (0.6,0.81)
130	Republic of Liberia	0	0.69 (0.39,0.99)
131	Republic of Zimbabwe	0	0.68 (0.59,0.77)
132	Bosnia and Herzegovina	0	0.67 (0.54,0.81)
133	Republic of Cote d'Ivoire	0	0.66 (0.58,0.73)
134	Burkina Faso	0	0.66 (0.61,0.71)
135	Togolese Republic	0	0.63 (0.57,0.69)
136	Republic of Cameroon	0	0.63 (0.54,0.72)
137	Republic of Croatia	0	0.62 (0.5,0.74)
138	People's Republic of Bangladesh	0	0.6 (0.56,0.63)
139	Georgia	0	0.59 (0.39,0.79)
140	Islamic Republic of Pakistan	0	0.58 (0.55,0.61)
141	Federated States of Micronesia	0	0.57 (0.49,0.65)
142	United Republic of Tanzania	0	0.56 (0.5,0.61)
143	Palestine	0	0.56 (0.44,0.68)
144	Montenegro	0	0.55 (0.4,0.7)
145	Canada	0	0.55 (0.44,0.65)
146	Republic of Tajikistan	0	0.54 (0.33,0.76)
147	Republic of Senegal	0	0.53 (0.5,0.56)
148	Tuvalu	0	0.51 (0.44,0.58)

149	Gabonese Republic	0	0.5 (0.47,0.53)
150	Republic of San Marino	0.002	0.49 (0.21,0.77)
151	Principality of Andorra	0	0.48 (0.33,0.64)
152	Republic of Vanuatu	0	0.46 (0.43,0.49)
153	Republic of Iceland	0	0.46 (0.4,0.53)
154	Republic of India	0	0.44 (0.39,0.5)
155	Cook Islands	0	0.43 (0.27,0.58)
156	Republic of Benin	0	0.43 (0.41,0.46)
157	Independent State of Samoa	0	0.42 (0.24,0.61)
158	Republic of Austria	0	0.42 (0.23,0.61)
159	Republic of Guinea-Bissau	0	0.41 (0.31,0.51)
160	Independent State of Papua New Guinea	0	0.4 (0.35,0.45)
161	Republic of the Gambia	0	0.4 (0.37,0.42)
162	Republic of Rwanda	0.016	0.4 (0.09,0.71)
163	French Republic	0	0.4 (0.32,0.49)
164	Republic of Malawi	0	0.38 (0.3,0.45)
165	Republic of Albania	0	0.38 (0.19,0.57)
166	Kyrgyz Republic	0.001	0.36 (0.17,0.55)
167	New Zealand	0.057	0.36 (0,0.73)
168	Federal Republic of Somalia	0.053	0.35 (0.01,0.69)
169	Republic of Lithuania	0	0.35 (0.23,0.47)
170	Islamic Republic of Mauritania	0	0.33 (0.3,0.36)
171	Republic of Uganda	0	0.33 (0.29,0.37)
172	American Samoa	0	0.32 (0.19,0.46)
173	Grand Duchy of Luxembourg	0	0.31 (0.2,0.42)

174	Kingdom of Norway	0	0.31 (0.2,0.42)
175	Federal Democratic Republic of Ethiopia	0	0.3 (0.21,0.39)
176	Swiss Confederation	0	0.28 (0.17,0.39)
177	Federal Democratic Republic of Nepal	0	0.27 (0.22,0.31)
178	Republic of Nauru	0	0.24 (0.17,0.31)
179	Kingdom of Belgium	0	0.24 (0.13,0.35)
180	Kingdom of Denmark	0.034	0.23 (0.03,0.44)
181	Republic of South Sudan	0	0.23 (0.14,0.32)
182	United Kingdom of Great Britain and Northern Ireland	0.024	0.23 (0.04,0.43)
183	Republic of Madagascar	0	0.22 (0.18,0.25)
184	Republic of Finland	0	0.22 (0.12,0.32)
185	Republic of Mozambique	0	0.22 (0.14,0.29)
186	Federal Republic of Germany	0.063	0.19 (0,0.39)
187	Republic of Kiribati	0.036	0.18 (0.02,0.34)
188	Republic of Angola	0	0.18 (0.11,0.24)
189	Democratic Republic of the Congo	0.001	0.12 (0.05,0.18)
190	Republic of Chad	0.002	0.06 (0.02,0.09)
191	Central African Republic	0.07	0.05 (0,0.1)
192	Republic of Burundi	0.692	0.05 (-0.19,0.29)
193	Republic of Mali	0.292	0.03 (-0.02,0.08)
194	Republic of Sierra Leone	0.588	0.02 (-0.06,0.1)
195	Guam	0.896	0.01 (-0.13,0.14)
196	Federal Republic of Nigeria	0.887	0 (-0.06,0.07)
197	Greenland	0.888	-0.02 (-0.36,0.31)
198	Republic of Guinea	0.504	-0.03 (-0.11,0.05)

199	Northern Mariana Islands	0.246	-0.19 (-0.52,0.13)
200	Principality of Monaco	0.107	-0.21 (-0.45,0.04)
201	Republic of the Niger	0	-0.25 (-0.36,-0.14)
202	Kingdom of the Netherlands	0.036	-0.32 (-0.6,-0.03)
203	Democratic Republic of Timor-Leste	0	-0.35 (-0.39,-0.31)
204	Kingdom of Sweden	0.003	-0.57 (-0.91,-0.23)

Supplementary Material 3

The detailed APCs and 95%CI of Joinpoint regression for each sex group

Annual Percent Change(APC) of ASPR							
**Cohort**	**Segment**	**Lower Endpoint**	**Upper Endpoint**	**APC**	**Lower CI**	**Upper CI**	**P Value**
Both	1	1990	1995	-0.4446*	-0.5494	-0.3398	0.000001
Both	2	1995	2000	0.8268*	0.6773	0.9764	< 0.000001
Both	3	2000	2005	1.3627*	1.214	1.5117	< 0.000001
Both	4	2005	2011	0.2246*	0.1213	0.328	0.000479
Both	5	2011	2015	0.6798*	0.4484	0.9118	0.000033
Both	6	2015	2019	0.205	-0.0279	0.4384	0.079291
Both	7	2019	2021	0.8657*	0.394	1.3396	0.001742
Female	1	1990	1993	0.3147*	0.2473	0.3822	< 0.000001
Female	2	1993	2001	0.0225*	0.0046	0.0405	0.018193
Female	3	2001	2005	0.5770*	0.5095	0.6446	< 0.000001
Female	4	2005	2009	1.4392*	1.3716	1.5068	< 0.000001
Female	5	2009	2014	0.4807*	0.439	0.5224	< 0.000001
Female	6	2014	2019	-0.0915*	-0.1334	-0.0495	0.000473
Female	7	2019	2021	0.6083*	0.4759	0.741	< 0.000001
Male	1	1990	1994	-0.9222*	-1.1256	-0.7183	< 0.000001
Male	2	1994	1997	0.4431	-0.2027	1.0931	0.161124
Male	3	1997	2005	1.6421*	1.5553	1.729	< 0.000001
Male	4	2005	2010	0.0667	-0.1345	0.2683	0.484037
Male	5	2010	2015	0.6090*	0.4087	0.8097	0.000024
Male	6	2015	2019	-0.3075	-0.6265	0.0124	0.058123
Male	7	2019	2021	0.4223	-0.2238	1.0726	0.180371

*indicates that the APC is significantly different from zero at the alpha = 0.05 level							
**Annual Percent Change(APC) of ASIR**							
**Cohort**	**Segment**	**Lower Endpoint**	**Upper Endpoint**	**APC**	**Lower CI**	**Upper CI**	**P Value**
Both	1	1990	1995	-0.4797*	-0.5740	-0.3853	< 0.000001
Both	2	1995	2000	0.8490*	0.7138	0.9844	< 0.000001
Both	3	2000	2005	1.3714*	1.2356	1.5074	< 0.000001
Both	4	2005	2011	0.2498*	0.1550	0.3447	0.000092
Both	5	2011	2015	0.7372*	0.5252	0.9497	0.000006
Both	6	2015	2019	0.1990	-0.0123	0.4106	0.062621
Both	7	2019	2021	0.8220*	0.3936	1.2522	0.001256
Female	1	1990	1994	0.3265*	0.2880	0.3649	< 0.000001
Female	2	1994	2001	0.0070	-0.0136	0.0275	0.473514
Female	3	2001	2005	0.6468*	0.5854	0.7083	< 0.000001
Female	4	2005	2010	1.3610*	1.3220	1.4001	< 0.000001
Female	5	2010	2015	0.2755*	0.2373	0.3138	< 0.000001
Female	6	2015	2019	-0.0850*	-0.1451	-0.0248	0.009637
Female	7	2019	2021	0.7078*	0.5844	0.8314	< 0.000001
Male	1	1990	1994	-0.9677*	-1.1635	-0.7715	< 0.000001
Male	2	1994	1997	0.4700	-0.1552	1.0990	0.127619
Male	3	1997	2005	1.6386*	1.5545	1.7228	< 0.000001
Male	4	2005	2010	0.0867	-0.1080	0.2819	0.351340
Male	5	2010	2015	0.6303*	0.4349	0.8260	0.000013
Male	6	2015	2019	-0.3143*	-0.6227	-0.0050	0.046969
Male	7	2019	2021	0.3775	-0.2491	1.0081	0.214399
*indicates that the APC is significantly different from zero at the alpha = 0.05 level							

Annual Percent Change(APC) of ASDR							
**Cohort**	**Segment**	**Lower Endpoint**	**Upper Endpoint**	**APC**	**Lower CI**	**Upper CI**	**P Value**
Both	1	1990	1994	-0.6673*	-1.1117	-0.2210	0.005351
Both	2	1994	2000	0.6305*	0.3100	0.9521	0.000515
Both	3	2000	2004	1.5587*	0.8516	2.2707	0.000154
Both	4	2004	2021	0.4275*	0.3778	0.4772	< 0.000001
Female	1	1990	1994	0.5180*	0.1422	0.8952	0.009192
Female	2	1994	2004	0.0790	-0.0284	0.1864	0.140988
Female	3	2004	2011	1.2557*	1.0513	1.4606	< 0.000001
Female	4	2011	2021	0.1440*	0.0525	0.2357	0.003639
Male	1	1990	1993	-1.1452*	-1.7791	-0.5073	0.001685
Male	2	1993	1996	-0.2009	-1.4797	1.0945	0.744229
Male	3	1996	2005	1.6258*	1.4851	1.7667	< 0.000001
Male	4	2005	2010	0.0294	-0.3625	0.4228	0.875294
Male	5	2010	2014	0.7634*	0.1268	1.4040	0.021859
Male	6	2014	2021	-0.0486	-0.2182	0.1212	0.550755
*indicates that the APC is significantly different from zero at the alpha = 0.05 level							

Supplementary Material 4

The differencing order and the selected (p, d, q) parameters determined by auto.arima, and the ACF, PACF plots, QQ plot, and Ljung-Box test results are as follows:

ASPR of Both

Differencing order: d=1   Selected (p, d, q) parameters: ARIMA(1,1,3)

```
ARIMA(1,1,3)

Coefficients:
         ar1     ma1     ma2     ma3
      0.7766  1.2184  1.2193  0.7935
s.e.  0.1165  0.2050  0.3501  0.2611

sigma^2 = 0.1697:  log likelihood = -17.91
AIC=45.83   AICc=48.23   BIC=53
```

ACF, PACF plots

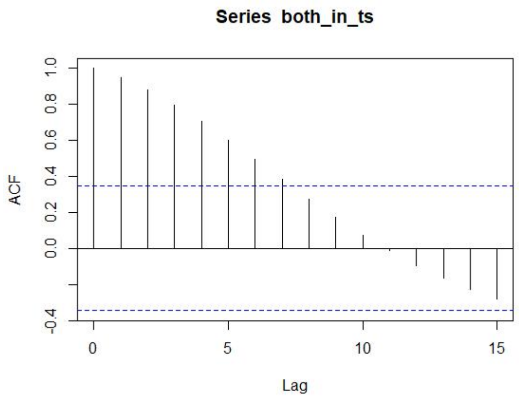

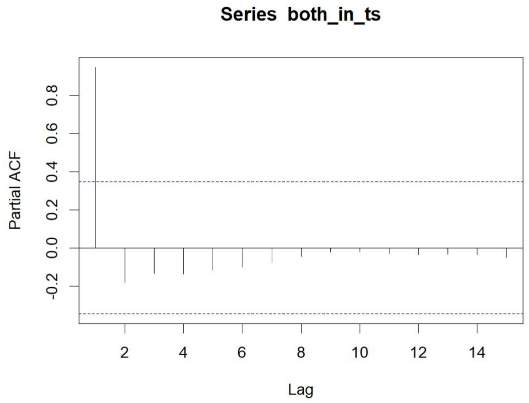

QQ plot

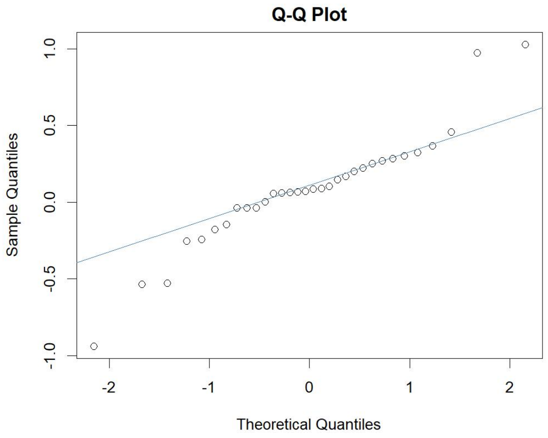

Box-Ljung test
data: residuals(arima_model)
X-squared = 13.972, df = 24, p-value = 0.9473

ASPR of Male

Differencing order: d=1   Selected (p, d, q) parameters: ARIMA(2,1,1)

```
ARIMA(2,1,1)

Coefficients:
         ar1      ar2     ma1
      1.5239  -0.6179  0.8842
s.e.  0.1410   0.1440  0.1141

sigma^2 = 0.4526:  log likelihood = -33.34
AIC=74.69   AICc=76.22   BIC=80.42
```

ACF, PACF plots

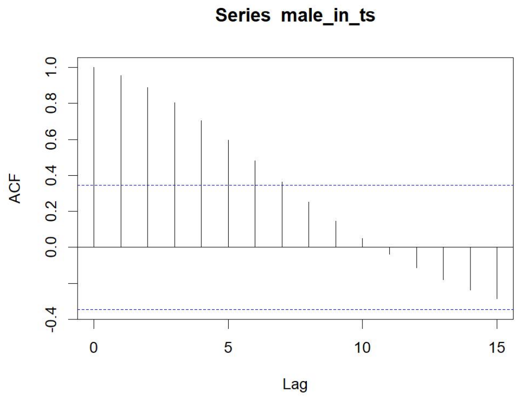

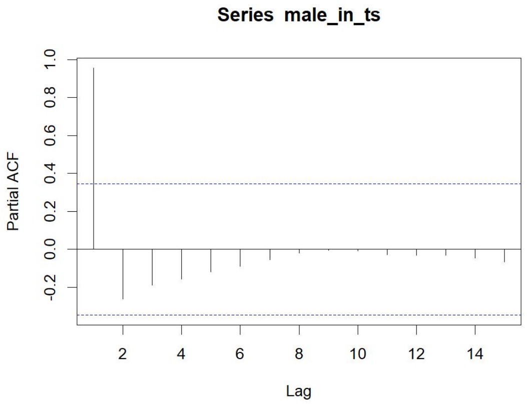

QQ plot

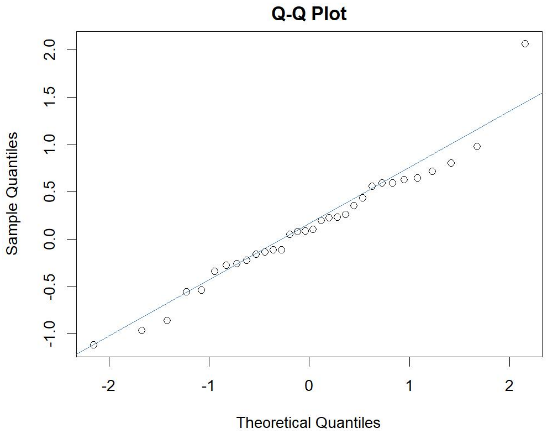

Box-Ljung test

data: residuals(arima_model)

X-squared = 18.603, df = 24, p-value = 0.7728

ASPR of Female

Differencing order: d=1   Selected (p, d, q) parameters: ARIMA(1,1,0)

```
ARIMA(1,1,0)

Coefficients:
         ar1
      0.9195
s.e.  0.0570

sigma^2 = 0.0486:  log likelihood = 2.54
AIC=-1.08   AICc=-0.65   BIC=1.79
```

ACF, PACF plots

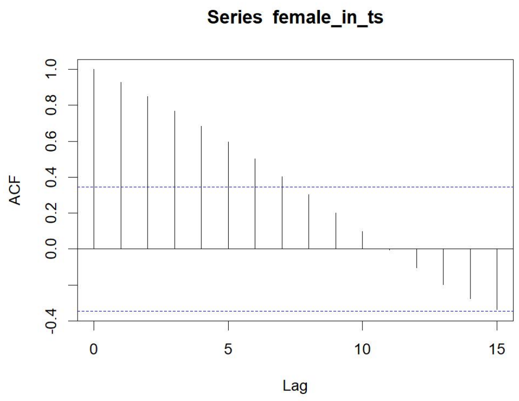

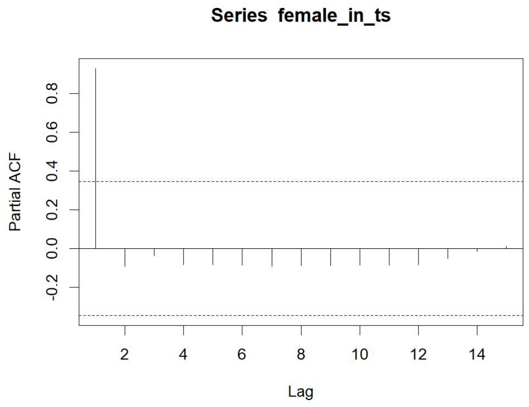

QQ plot

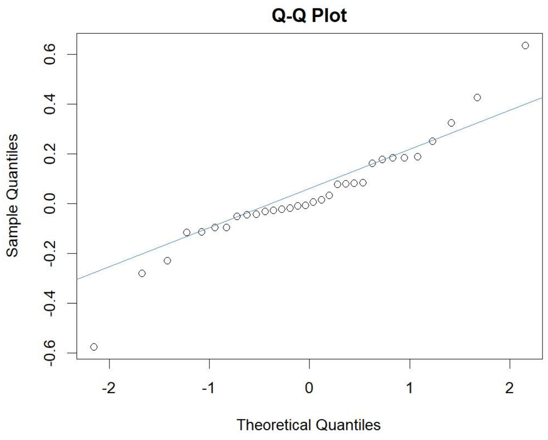

Box-Ljung test

data: residuals(arima_model)

X-squared = 14.576, df = 24, p-value = 0.9325

ASIR of Both

Differencing order: d=1   Selected (p, d, q) parameters: ARIMA(2,1,0)

```
ARIMA(2,1,0)

Coefficients:
         ar1      ar2
      1.5021  -0.5869
s.e.  0.1530   0.1572

sigma^2 = 0.02359:  log likelihood = 13.65
AIC=-21.29   AICc=-20.4   BIC=-16.99
```

ACF, PACF plots

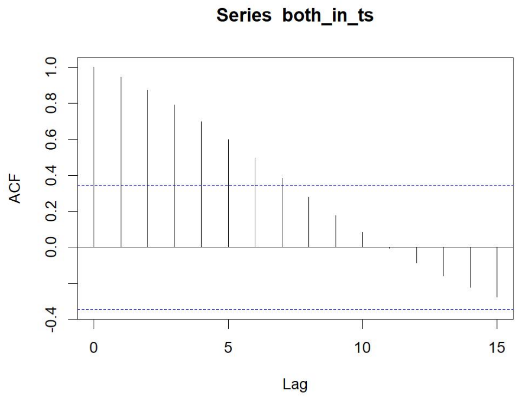

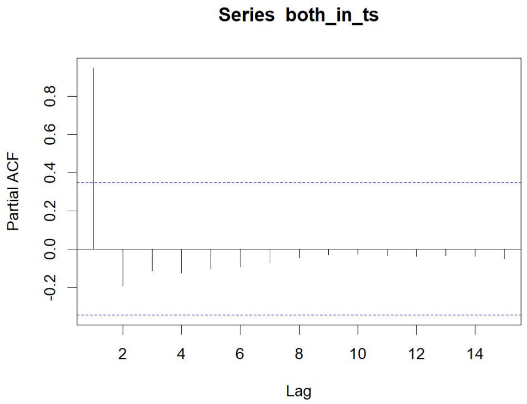

QQ plot

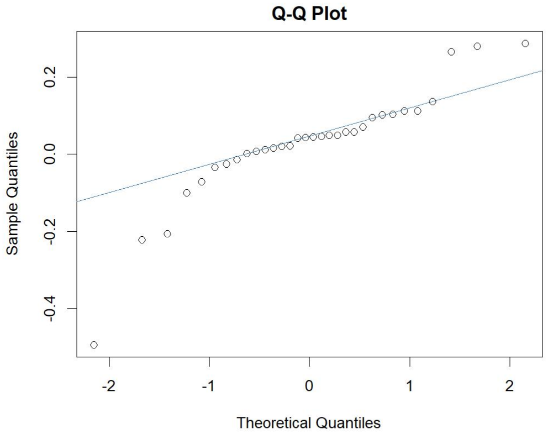

Box-Ljung test

data: residuals(arima_model)

X-squared = 13.504, df = 24, p-value = 0.9571

ASIR of Male

Differencing order: d=1   Selected (p, d, q) parameters: ARIMA(2,1,0)

```
ARIMA(2,1,0)

Coefficients:
         ar1      ar2
      1.6059  -0.6939
s.e.  0.1295   0.1320

sigma^2 = 0.07195:  log likelihood = -3.9
AIC=13.8   AICc=14.69   BIC=18.1
```

ACF, PACF plots

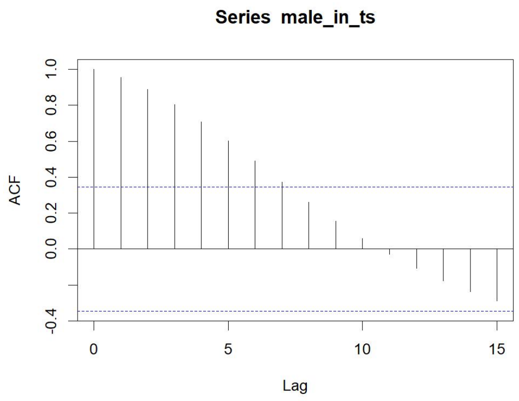

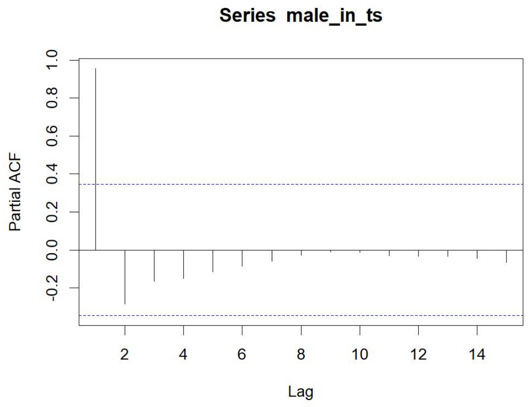

QQ plot

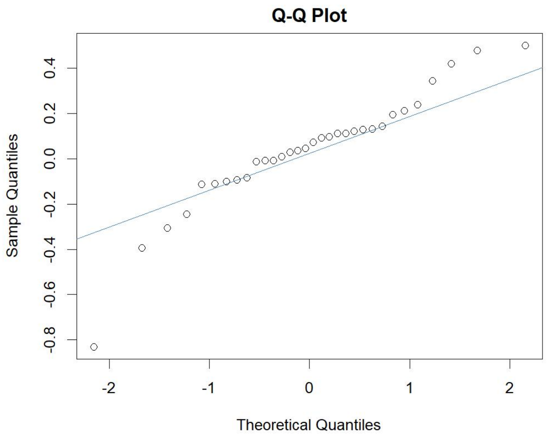

Box-Ljung test
data: residuals(arima_model)

X-squared = 15.285, df = 24, p-value = 0.9121

ASIR of Female

Differencing order: d=2   Selected (p, d, q) parameters: ARIMA(0,2,0)

```
ARIMA(0,2,0)

sigma^2 = 0.002852:  log likelihood = 45.5
AIC=-88.99   AICc=-88.85   BIC=-87.59
```

ACF, PACF plots

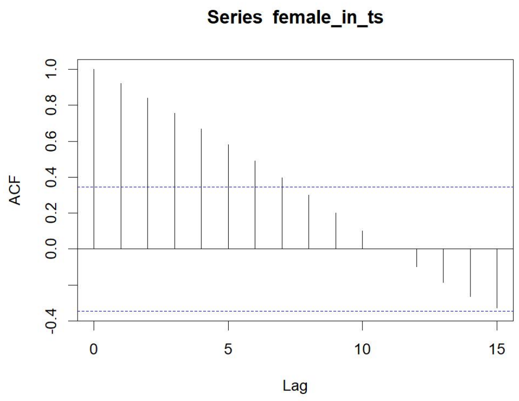

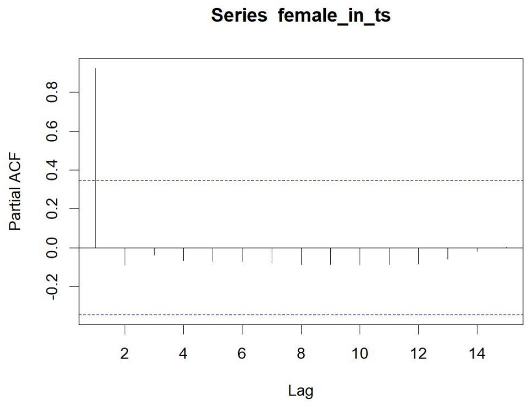

QQ plot

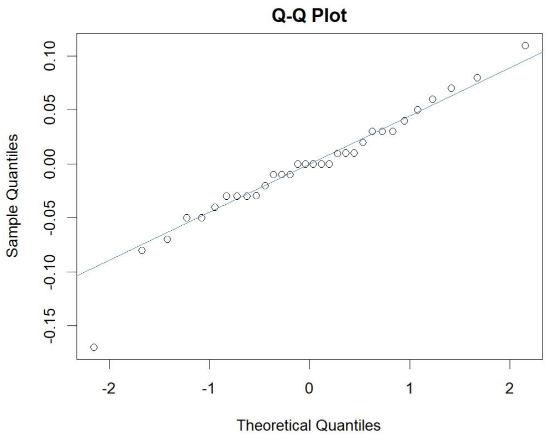

Box-Ljung test

data: residuals(arima_model)

X-squared = 15.425, df = 24, p-value = 0.9077

ASDR of Both

Differencing order: d=1   Selected (p, d, q) parameters: ARIMA(1,1,2)

```
ARIMA(1,1,2)

Coefficients:
         ar1     ma1     ma2
      0.6989  0.1691  0.7063
s.e.  0.1771  0.1806  0.2389

sigma^2 = 0.001381:  log likelihood = 58.27
AIC=-108.54   AICc=-107   BIC=-102.8
```

ACF, PACF plots

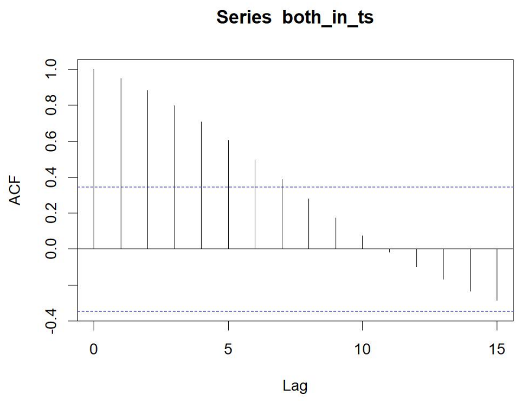

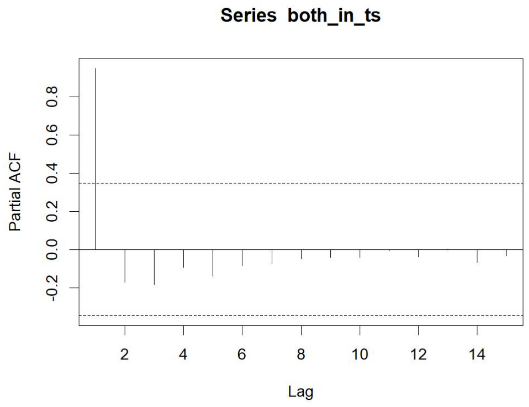

QQ plot

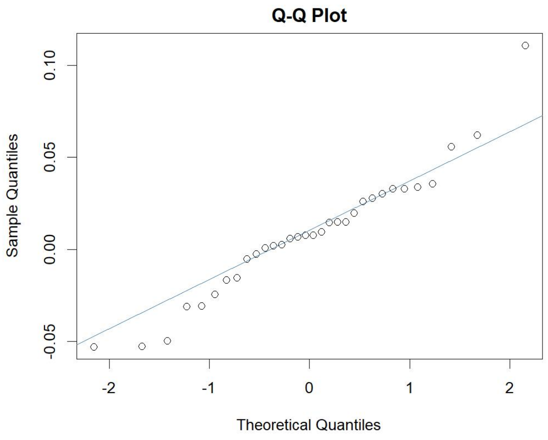

Box-Ljung test

data: residuals(arima_model)

X-squared = 20.566, df = 24, p-value = 0.6641

ASDR of Male

Differencing order: d=1   Selected (p, d, q) parameters: ARIMA(1,1,2)

```
ARIMA(1,1,2)

Coefficients:
         ar1     ma1     ma2
      0.7962  0.3540  0.5909
s.e.  0.1459  0.2695  0.1764

sigma^2 = 0.003275:  log likelihood = 44.84
AIC=-81.67   AICc=-80.13   BIC=-75.94
```

ACF, PACF plots

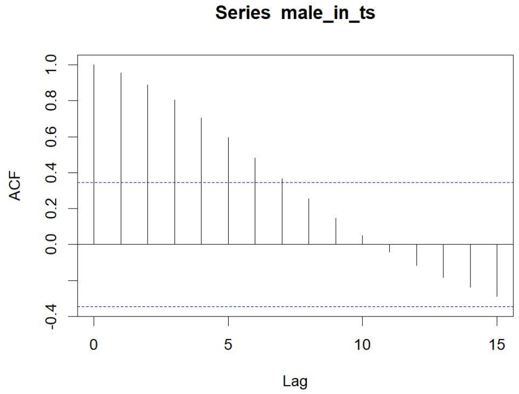

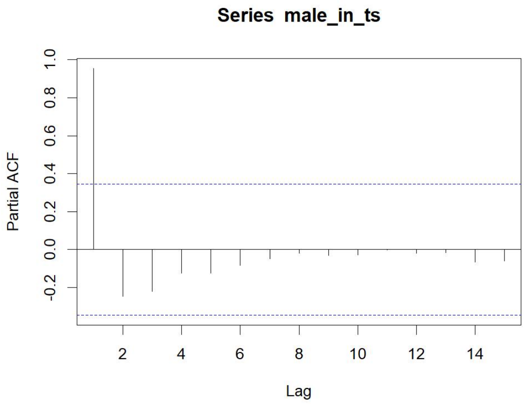

QQ plot

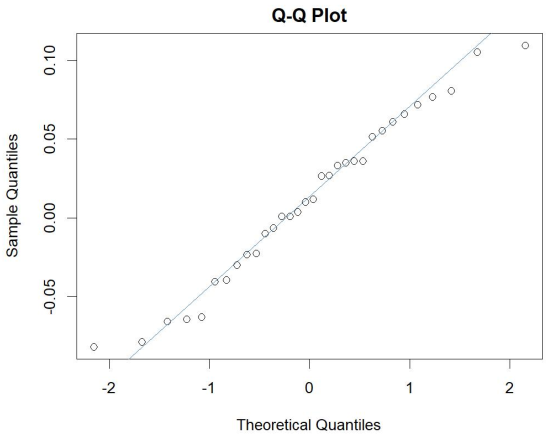

Box-Ljung test
data: residuals(arima_model)

X-squared = 20.487, df = 24, p-value = 0.6688

ASDR of Female

Differencing order: d=1   Selected (p, d, q) parameters: ARIMA(0,2,0)

```
ARIMA(1,1,0) with drift

Coefficients:
         ar1   drift
      0.3366  0.0147
s.e.  0.1673  0.0045

sigma^2 = 0.0003098:  log likelihood = 82.23
AIC=-158.47   AICc=-157.58   BIC=-154.17
```

ACF, PACF plots

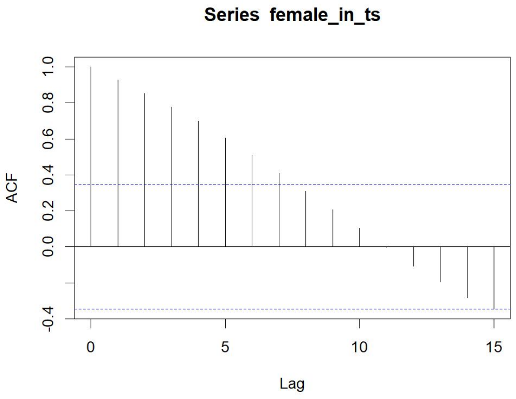

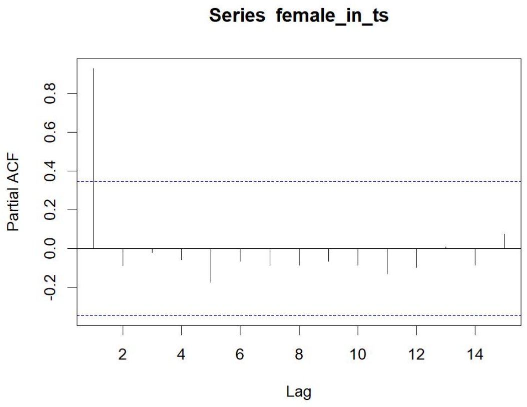

QQ plot

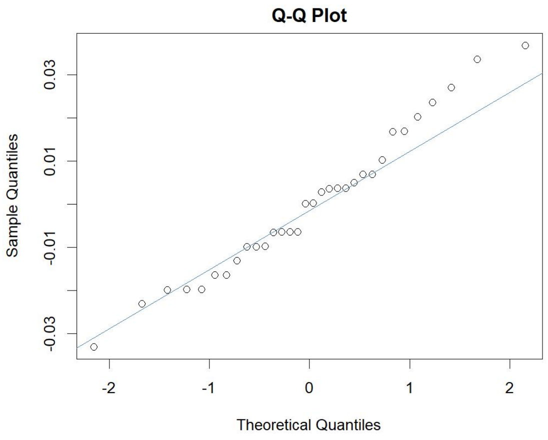

Box-Ljung test

data: residuals(arima_model)

X-squared = 24.695, df = 24, p-value = 0.4225

Supplementary Material 5

Sensitive Analysis of ARIMA model

1.Parameter Sensitivity

we evaluated the performance discrepancies of ARIMA model for ASIR under various combinations of (p, d, q) parameters. The predicted values exhibited variations corresponding to the different selected parameters, the black points represent the value predicted by auto.arima function. Consequently, the model's performance is significantly contingent upon the precision of parameter selection. To achieve enhanced predictive accuracy, we employed the auto.arima function to automatically fit the (p, d, q) parameters, thereby seeking to minimize the AIC and BIC values.

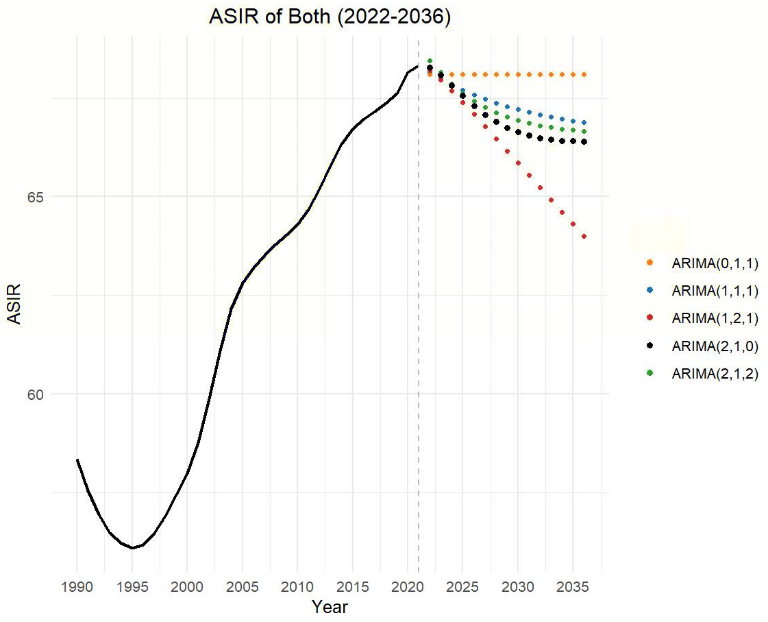

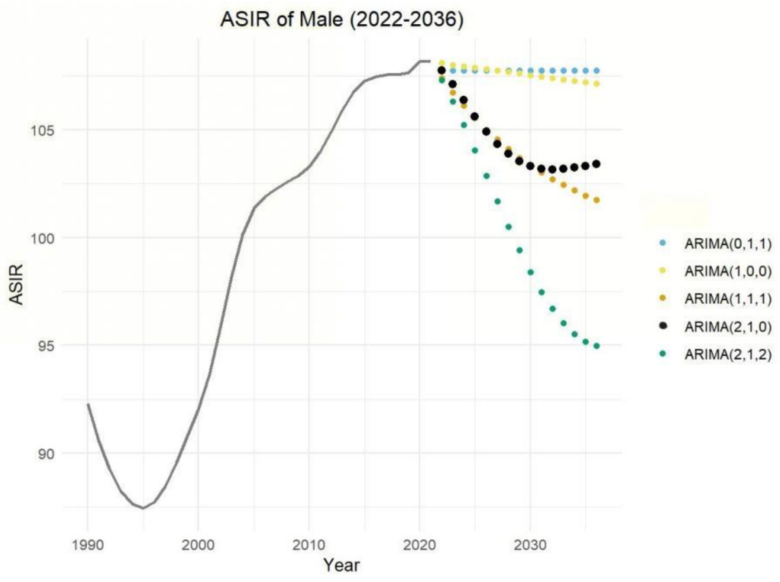

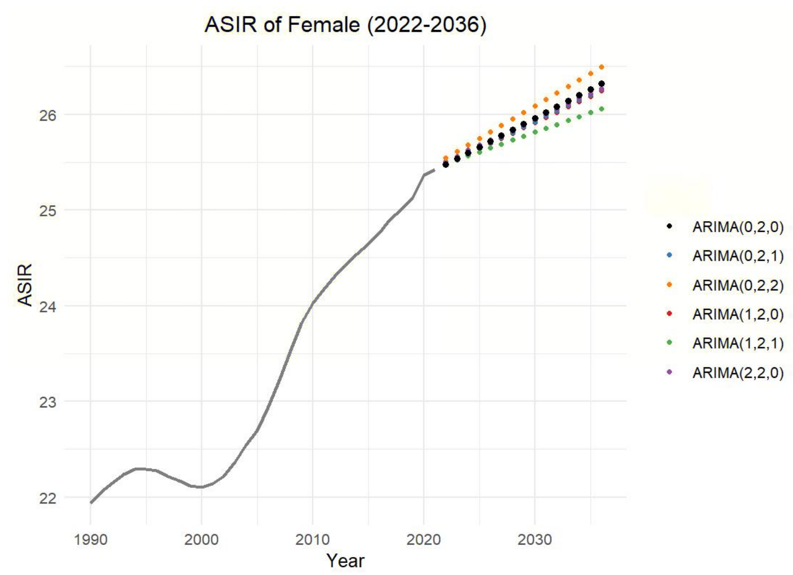

2.Data Variation Sensitivity

The ARIMA model is sensitive to data changes and missing values. We used the provided data from 1990 to 2018 to reforecast the future in order to assess whether data fluctuations since the COVID-19 era would affect the predictive performance of the ARIMA model. It can be concluded that the **ASIR of Both** and **ASDR of Both** showed significant trend differences after removing the data from 2019 to 2021.

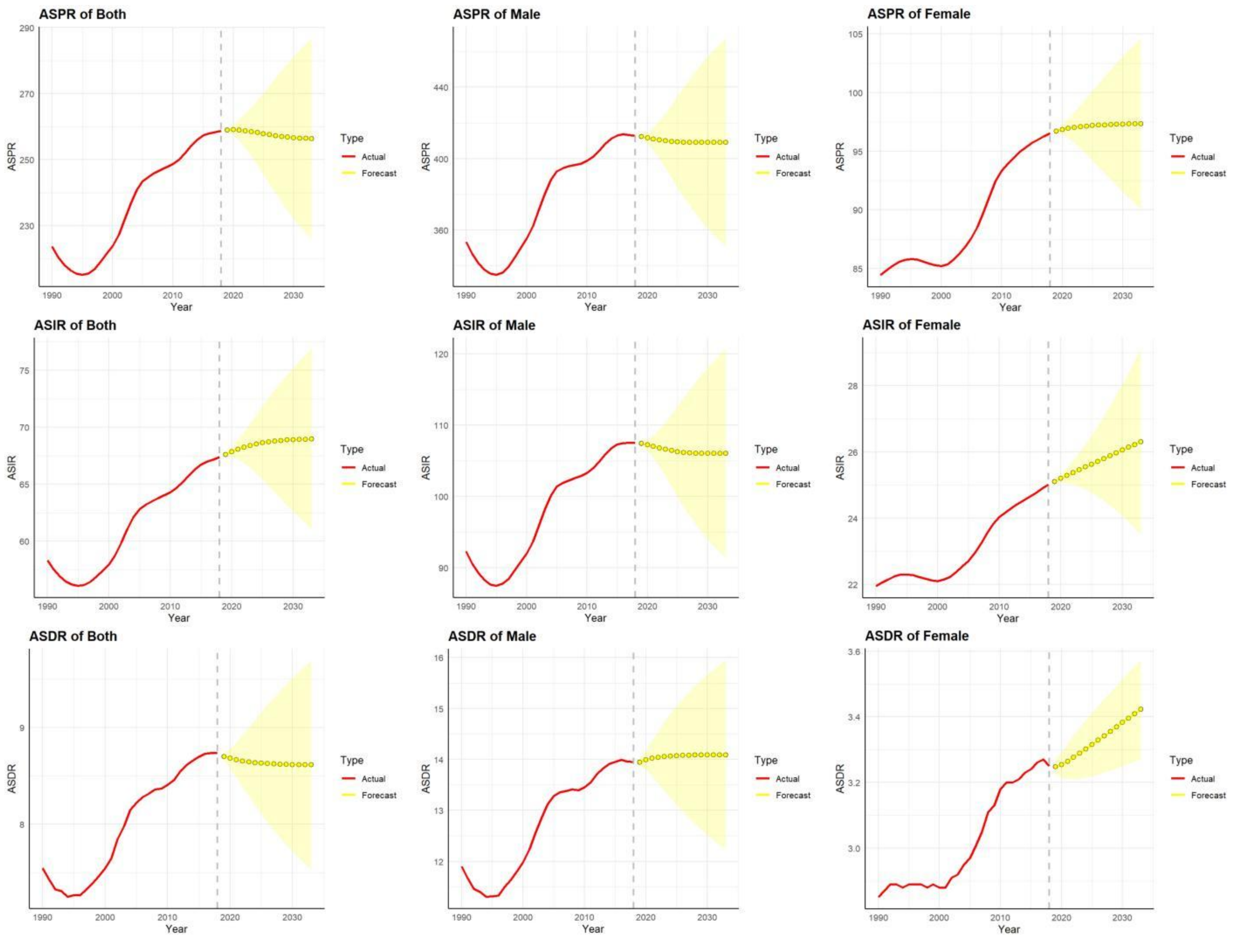

Supplementary Material 6

The detailed exposure values and 95% CI for each risk factor and each sex group in China's 15-39 years cohort were listed as followed.

Sex	Rei name	year	val	upper	lower
Female	Hypertension	1990	11.88	30.16	1.68
Female	Hypertension	1991	12.13	30.94	1.72
Female	Hypertension	1992	12.45	31.92	1.80
Female	Hypertension	1993	12.74	32.54	1.88
Female	Hypertension	1994	13.11	33.50	1.92
Female	Hypertension	1995	13.46	34.70	1.92
Female	Hypertension	1996	13.87	35.74	2.13
Female	Hypertension	1997	14.28	37.02	2.26
Female	Hypertension	1998	14.75	37.93	2.30
Female	Hypertension	1999	15.19	38.52	2.49
Female	Hypertension	2000	15.64	39.02	2.73
Female	Hypertension	2001	16.13	39.48	2.91
Female	Hypertension	2002	16.61	40.08	3.10
Female	Hypertension	2003	17.07	40.94	3.30
Female	Hypertension	2004	17.52	42.36	3.30
Female	Hypertension	2005	18.06	43.78	3.34
Female	Hypertension	2006	18.60	43.86	3.60
Female	Hypertension	2007	18.93	44.23	3.75
Female	Hypertension	2008	19.20	45.17	3.80
Female	Hypertension	2009	19.40	44.88	3.55
Female	Hypertension	2010	19.74	45.81	3.54
Female	Hypertension	2011	19.75	45.57	3.62

Female	Hypertension	2012	19.77	44.67	3.67
Female	Hypertension	2013	19.85	43.85	3.75
Female	Hypertension	2014	19.92	44.52	3.97
Female	Hypertension	2015	20.02	45.80	3.95
Female	Hypertension	2016	20.19	46.51	4.26
Female	Hypertension	2017	20.44	46.67	4.67
Female	Hypertension	2018	20.70	46.80	4.88
Female	Hypertension	2019	20.96	46.79	5.14
Female	Hypertension	2020	21.17	46.62	5.25
Female	Hypertension	2021	21.37	46.36	5.30
Male	Hypertension	1990	16.79	37.26	4.82
Male	Hypertension	1991	17.38	38.08	5.06
Male	Hypertension	1992	18.08	39.28	5.23
Male	Hypertension	1993	18.73	40.56	5.38
Male	Hypertension	1994	19.46	41.49	5.87
Male	Hypertension	1995	20.16	42.32	6.26
Male	Hypertension	1996	20.85	42.85	6.34
Male	Hypertension	1997	21.52	44.02	6.50
Male	Hypertension	1998	22.21	45.74	6.46
Male	Hypertension	1999	22.85	46.48	6.59
Male	Hypertension	2000	23.47	46.76	7.02
Male	Hypertension	2001	24.15	47.68	7.43
Male	Hypertension	2002	24.78	48.34	7.30
Male	Hypertension	2003	25.57	49.18	8.00
Male	Hypertension	2004	26.33	50.19	8.50

Male	Hypertension	2005	27.20	51.27	8.97
Male	Hypertension	2006	28.04	52.44	9.34
Male	Hypertension	2007	28.65	52.94	9.54
Male	Hypertension	2008	29.17	53.44	9.71
Male	Hypertension	2009	29.62	54.17	9.96
Male	Hypertension	2010	30.22	54.96	10.34
Male	Hypertension	2011	30.39	55.22	10.59
Male	Hypertension	2012	30.58	55.76	10.50
Male	Hypertension	2013	30.86	56.33	10.92
Male	Hypertension	2014	31.04	56.95	11.06
Male	Hypertension	2015	31.20	57.15	11.35
Male	Hypertension	2016	31.39	57.78	11.28
Male	Hypertension	2017	31.68	57.40	11.43
Male	Hypertension	2018	32.01	57.19	11.94
Male	Hypertension	2019	32.30	56.44	12.18
Male	Hypertension	2020	32.56	55.89	12.31
Male	Hypertension	2021	32.80	55.74	12.54

Sex	Rei name	year	val	upper	lower
Female	High Fasting Glucose	1990	10.94	15.40	7.19
Female	High Fasting Glucose	1991	10.92	15.50	7.14
Female	High Fasting Glucose	1992	10.90	15.49	7.05
Female	High Fasting Glucose	1993	10.90	15.33	6.98
Female	High Fasting Glucose	1994	10.90	15.44	6.95
Female	High Fasting Glucose	1995	10.98	15.56	6.94

Female	High Fasting Glucose	1996	11.11	15.67	7.09
Female	High Fasting Glucose	1997	11.29	15.96	7.17
Female	High Fasting Glucose	1998	11.51	16.30	7.36
Female	High Fasting Glucose	1999	11.73	16.40	7.49
Female	High Fasting Glucose	2000	11.92	16.61	7.73
Female	High Fasting Glucose	2001	12.11	16.89	7.87
Female	High Fasting Glucose	2002	12.30	17.02	8.05
Female	High Fasting Glucose	2003	12.65	17.53	8.25
Female	High Fasting Glucose	2004	13.00	17.87	8.47
Female	High Fasting Glucose	2005	13.31	18.24	8.67
Female	High Fasting Glucose	2006	13.61	18.48	8.78
Female	High Fasting Glucose	2007	13.90	19.10	8.91
Female	High Fasting Glucose	2008	14.18	19.68	9.15
Female	High Fasting Glucose	2009	14.47	20.20	9.44
Female	High Fasting Glucose	2010	14.77	20.52	9.68
Female	High Fasting Glucose	2011	14.92	20.64	9.81
Female	High Fasting Glucose	2012	15.07	20.83	9.97
Female	High Fasting Glucose	2013	15.28	21.13	10.19
Female	High Fasting Glucose	2014	15.42	21.65	10.34
Female	High Fasting Glucose	2015	15.54	21.98	10.43
Female	High Fasting Glucose	2016	15.70	21.97	10.55
Female	High Fasting Glucose	2017	15.98	21.77	10.67
Female	High Fasting Glucose	2018	16.28	21.80	10.91
Female	High Fasting Glucose	2019	16.50	22.53	11.01
Female	High Fasting Glucose	2020	16.68	22.71	11.11

Female	High Fasting Glucose	2021	16.98	22.90	11.21
Male	High Fasting Glucose	1990	14.56	20.65	10.11
Male	High Fasting Glucose	1991	14.49	20.77	9.93
Male	High Fasting Glucose	1992	14.45	20.75	9.88
Male	High Fasting Glucose	1993	14.44	20.50	9.82
Male	High Fasting Glucose	1994	14.42	20.57	9.93
Male	High Fasting Glucose	1995	14.51	21.04	9.99
Male	High Fasting Glucose	1996	14.67	21.45	10.16
Male	High Fasting Glucose	1997	14.92	21.76	10.27
Male	High Fasting Glucose	1998	15.22	21.55	10.59
Male	High Fasting Glucose	1999	15.53	21.51	10.86
Male	High Fasting Glucose	2000	15.82	21.37	11.18
Male	High Fasting Glucose	2001	16.08	21.26	11.48
Male	High Fasting Glucose	2002	16.37	21.71	11.75
Male	High Fasting Glucose	2003	16.83	22.04	12.12
Male	High Fasting Glucose	2004	17.29	22.63	12.46
Male	High Fasting Glucose	2005	17.72	23.23	12.77
Male	High Fasting Glucose	2006	18.12	23.69	13.08
Male	High Fasting Glucose	2007	18.51	23.98	13.44
Male	High Fasting Glucose	2008	18.88	24.24	13.66
Male	High Fasting Glucose	2009	19.25	24.80	14.03
Male	High Fasting Glucose	2010	19.66	25.45	14.39
Male	High Fasting Glucose	2011	19.78	25.60	14.57
Male	High Fasting Glucose	2012	19.89	25.73	14.45
Male	High Fasting Glucose	2013	20.05	26.39	14.45

Male	High Fasting Glucose	2014	20.31	26.69	14.54
Male	High Fasting Glucose	2015	20.53	26.88	14.61
Male	High Fasting Glucose	2016	20.79	27.61	14.76
Male	High Fasting Glucose	2017	21.25	28.22	15.14
Male	High Fasting Glucose	2018	21.73	28.65	15.64
Male	High Fasting Glucose	2019	22.10	29.46	16.06
Male	High Fasting Glucose	2020	22.40	29.69	16.17
Male	High Fasting Glucose	2021	22.77	29.93	16.40

Sex	Rei name	year	val	upper	lower
Female	High BMI	1990	6.38	8.42	5.03
Female	High BMI	1991	6.50	8.57	5.13
Female	High BMI	1992	6.62	8.74	5.24
Female	High BMI	1993	6.75	8.91	5.36
Female	High BMI	1994	6.88	9.07	5.49
Female	High BMI	1995	7.01	9.23	5.62
Female	High BMI	1996	7.15	9.39	5.76
Female	High BMI	1997	7.31	9.57	5.91
Female	High BMI	1998	7.48	9.76	6.08
Female	High BMI	1999	7.67	9.96	6.26
Female	High BMI	2000	7.86	10.15	6.45
Female	High BMI	2001	8.06	10.33	6.64
Female	High BMI	2002	8.26	10.52	6.85
Female	High BMI	2003	8.47	10.72	7.06
Female	High BMI	2004	8.69	10.87	7.29

Female	High BMI	2005	8.92	11.11	7.52
Female	High BMI	2006	9.15	11.39	7.76
Female	High BMI	2007	9.40	11.73	8.01
Female	High BMI	2008	9.67	12.11	8.25
Female	High BMI	2009	9.95	12.45	8.53
Female	High BMI	2010	10.24	12.80	8.81
Female	High BMI	2011	10.56	13.20	9.09
Female	High BMI	2012	10.90	13.59	9.40
Female	High BMI	2013	11.28	14.01	9.75
Female	High BMI	2014	11.68	14.49	10.14
Female	High BMI	2015	12.10	14.95	10.54
Female	High BMI	2016	12.54	15.49	10.97
Female	High BMI	2017	13.01	15.97	11.40
Female	High BMI	2018	13.50	16.52	11.86
Female	High BMI	2019	14.02	17.11	12.32
Female	High BMI	2020	14.57	17.69	12.79
Female	High BMI	2021	15.13	18.38	13.30
Male	High BMI	1990	7.36	9.41	5.99
Male	High BMI	1991	7.53	9.63	6.13
Male	High BMI	1992	7.72	9.87	6.30
Male	High BMI	1993	7.92	10.10	6.49
Male	High BMI	1994	8.12	10.35	6.71
Male	High BMI	1995	8.33	10.60	6.94
Male	High BMI	1996	8.56	10.85	7.15
Male	High BMI	1997	8.80	11.12	7.38

Male	High BMI	1998	9.06	11.39	7.64
Male	High BMI	1999	9.34	11.66	7.91
Male	High BMI	2000	9.61	11.93	8.19
Male	High BMI	2001	9.90	12.21	8.48
Male	High BMI	2002	10.20	12.50	8.80
Male	High BMI	2003	10.50	12.79	9.10
Male	High BMI	2004	10.82	13.12	9.43
Male	High BMI	2005	11.14	13.47	9.74
Male	High BMI	2006	11.48	13.86	10.04
Male	High BMI	2007	11.83	14.29	10.38
Male	High BMI	2008	12.19	14.72	10.74
Male	High BMI	2009	12.57	15.15	11.09
Male	High BMI	2010	12.94	15.52	11.46
Male	High BMI	2011	13.33	16.01	11.82
Male	High BMI	2012	13.75	16.47	12.25
Male	High BMI	2013	14.19	16.97	12.69
Male	High BMI	2014	14.67	17.53	13.10
Male	High BMI	2015	15.16	18.12	13.52
Male	High BMI	2016	15.66	18.73	13.98
Male	High BMI	2017	16.18	19.36	14.39
Male	High BMI	2018	16.72	20.02	14.88
Male	High BMI	2019	17.26	20.67	15.24
Male	High BMI	2020	17.81	21.28	15.66
Male	High BMI	2021	18.36	21.91	16.09

Sex	Rei name	year	val	upper	lower
Female	High Alcohol Use	1990	3.72	7.94	2.11
Female	High Alcohol Use	1991	3.78	8.13	2.19
Female	High Alcohol Use	1992	3.83	8.30	2.22
Female	High Alcohol Use	1993	3.87	8.41	2.26
Female	High Alcohol Use	1994	3.89	8.38	2.29
Female	High Alcohol Use	1995	3.91	8.33	2.31
Female	High Alcohol Use	1996	3.88	8.25	2.30
Female	High Alcohol Use	1997	3.81	8.16	2.24
Female	High Alcohol Use	1998	3.72	7.83	2.15
Female	High Alcohol Use	1999	3.64	7.50	2.10
Female	High Alcohol Use	2000	3.60	7.38	2.08
Female	High Alcohol Use	2001	3.60	7.43	2.10
Female	High Alcohol Use	2002	3.59	7.48	2.09
Female	High Alcohol Use	2003	3.59	7.43	2.08
Female	High Alcohol Use	2004	3.59	7.38	2.08
Female	High Alcohol Use	2005	3.61	7.35	2.10
Female	High Alcohol Use	2006	3.69	7.50	2.16
Female	High Alcohol Use	2007	3.85	7.81	2.26
Female	High Alcohol Use	2008	4.05	8.20	2.40
Female	High Alcohol Use	2009	4.25	8.54	2.53
Female	High Alcohol Use	2010	4.39	8.74	2.63
Female	High Alcohol Use	2011	4.50	8.85	2.71
Female	High Alcohol Use	2012	4.60	8.96	2.77
Female	High Alcohol Use	2013	4.70	9.12	2.80

Female	High Alcohol Use	2014	4.79	9.26	2.78
Female	High Alcohol Use	2015	4.86	9.29	2.79
Female	High Alcohol Use	2016	4.91	9.34	2.86
Female	High Alcohol Use	2017	4.95	9.47	2.92
Female	High Alcohol Use	2018	4.98	9.58	2.96
Female	High Alcohol Use	2019	5.02	9.59	3.01
Female	High Alcohol Use	2020	5.08	9.63	3.04
Female	High Alcohol Use	2021	5.11	9.64	3.10
Male	High Alcohol Use	1990	12.98	22.40	8.79
Male	High Alcohol Use	1991	13.20	22.54	8.98
Male	High Alcohol Use	1992	13.37	22.69	9.11
Male	High Alcohol Use	1993	13.50	22.76	9.17
Male	High Alcohol Use	1994	13.57	22.76	9.22
Male	High Alcohol Use	1995	13.61	22.72	9.25
Male	High Alcohol Use	1996	13.49	22.50	9.12
Male	High Alcohol Use	1997	13.18	22.00	8.85
Male	High Alcohol Use	1998	12.80	21.51	8.54
Male	High Alcohol Use	1999	12.46	21.15	8.30
Male	High Alcohol Use	2000	12.29	21.02	8.13
Male	High Alcohol Use	2001	12.22	21.05	8.16
Male	High Alcohol Use	2002	12.17	21.11	8.16
Male	High Alcohol Use	2003	12.13	20.93	8.18
Male	High Alcohol Use	2004	12.12	20.77	8.18
Male	High Alcohol Use	2005	12.15	20.68	8.18
Male	High Alcohol Use	2006	12.37	20.95	8.41

Male	High Alcohol Use	2007	12.86	21.57	8.81
Male	High Alcohol Use	2008	13.49	22.35	9.28
Male	High Alcohol Use	2009	14.10	23.15	9.75
Male	High Alcohol Use	2010	14.57	23.91	10.14
Male	High Alcohol Use	2011	14.91	24.49	10.43
Male	High Alcohol Use	2012	15.26	25.05	10.72
Male	High Alcohol Use	2013	15.57	25.44	10.94
Male	High Alcohol Use	2014	15.82	25.73	11.14
Male	High Alcohol Use	2015	16.01	25.95	11.30
Male	High Alcohol Use	2016	16.13	26.06	11.47
Male	High Alcohol Use	2017	16.22	26.20	11.56
Male	High Alcohol Use	2018	16.29	26.27	11.61
Male	High Alcohol Use	2019	16.36	26.33	11.60
Male	High Alcohol Use	2020	16.48	26.32	11.72
Male	High Alcohol Use	2021	16.54	26.31	11.76

Sex	Rei name	year	val	upper	lower
Female	High Processed Meat Intake	1990	2.61	3.30	1.52
Female	High Processed Meat Intake	1991	2.61	3.24	1.51
Female	High Processed Meat Intake	1992	2.61	3.22	1.52
Female	High Processed Meat Intake	1993	2.62	3.23	1.54
Female	High Processed Meat Intake	1994	2.64	3.21	1.56
Female	High Processed Meat Intake	1995	2.67	3.23	1.59
Female	High Processed Meat Intake	1996	2.71	3.26	1.63
Female	High Processed Meat Intake	1997	2.76	3.28	1.66

Female	High Processed Meat Intake	1998	2.81	3.31	1.69
Female	High Processed Meat Intake	1999	2.88	3.36	1.75
Female	High Processed Meat Intake	2000	2.95	3.43	1.83
Female	High Processed Meat Intake	2001	3.05	3.52	1.92
Female	High Processed Meat Intake	2002	3.18	3.64	2.02
Female	High Processed Meat Intake	2003	3.33	3.80	2.12
Female	High Processed Meat Intake	2004	3.52	3.98	2.25
Female	High Processed Meat Intake	2005	3.74	4.20	2.42
Female	High Processed Meat Intake	2006	3.98	4.44	2.61
Female	High Processed Meat Intake	2007	4.27	4.74	2.81
Female	High Processed Meat Intake	2008	4.56	5.06	3.02
Female	High Processed Meat Intake	2009	4.88	5.40	3.25
Female	High Processed Meat Intake	2010	5.20	5.73	3.50
Female	High Processed Meat Intake	2011	5.50	6.10	3.72
Female	High Processed Meat Intake	2012	5.77	6.50	3.92
Female	High Processed Meat Intake	2013	6.00	6.80	4.09
Female	High Processed Meat Intake	2014	6.20	7.15	4.24
Female	High Processed Meat Intake	2015	6.38	7.47	4.37
Female	High Processed Meat Intake	2016	6.53	7.73	4.56
Female	High Processed Meat Intake	2017	6.66	7.89	4.67
Female	High Processed Meat Intake	2018	6.76	8.09	4.73
Female	High Processed Meat Intake	2019	6.84	8.26	4.71
Female	High Processed Meat Intake	2020	6.88	8.42	4.64
Female	High Processed Meat Intake	2021	6.87	8.55	4.71
Male	High Processed Meat Intake	1990	2.42	3.06	1.50

Male	High Processed Meat Intake	1991	2.41	3.00	1.48
Male	High Processed Meat Intake	1992	2.41	2.97	1.46
Male	High Processed Meat Intake	1993	2.42	2.95	1.47
Male	High Processed Meat Intake	1994	2.43	2.96	1.47
Male	High Processed Meat Intake	1995	2.45	2.98	1.48
Male	High Processed Meat Intake	1996	2.48	2.97	1.50
Male	High Processed Meat Intake	1997	2.51	2.98	1.53
Male	High Processed Meat Intake	1998	2.55	3.01	1.56
Male	High Processed Meat Intake	1999	2.61	3.05	1.60
Male	High Processed Meat Intake	2000	2.67	3.12	1.65
Male	High Processed Meat Intake	2001	2.75	3.18	1.71
Male	High Processed Meat Intake	2002	2.85	3.28	1.79
Male	High Processed Meat Intake	2003	2.98	3.42	1.86
Male	High Processed Meat Intake	2004	3.14	3.56	1.97
Male	High Processed Meat Intake	2005	3.32	3.75	2.10
Male	High Processed Meat Intake	2006	3.53	3.97	2.26
Male	High Processed Meat Intake	2007	3.76	4.22	2.42
Male	High Processed Meat Intake	2008	4.01	4.49	2.60
Male	High Processed Meat Intake	2009	4.27	4.76	2.81
Male	High Processed Meat Intake	2010	4.53	5.06	3.02
Male	High Processed Meat Intake	2011	4.76	5.35	3.20
Male	High Processed Meat Intake	2012	4.97	5.66	3.36
Male	High Processed Meat Intake	2013	5.15	5.92	3.48
Male	High Processed Meat Intake	2014	5.31	6.14	3.58
Male	High Processed Meat Intake	2015	5.44	6.34	3.70

Male	High Processed Meat Intake	2016	5.56	6.58	3.78
Male	High Processed Meat Intake	2017	5.66	6.78	3.83
Male	High Processed Meat Intake	2018	5.74	7.03	3.89
Male	High Processed Meat Intake	2019	5.80	7.17	3.95
Male	High Processed Meat Intake	2020	5.83	7.27	3.97
Male	High Processed Meat Intake	2021	5.82	7.27	3.97

Sex	Rei name	year	val	upper	lower
Female	High Sugar-Sweetened Beverages Intake	1990	1.33	1.90	0.90
Female	High Sugar-Sweetened Beverages Intake	1991	1.34	1.86	0.91
Female	High Sugar-Sweetened Beverages Intake	1992	1.36	1.85	0.96
Female	High Sugar-Sweetened Beverages Intake	1993	1.39	1.85	1.00
Female	High Sugar-Sweetened Beverages Intake	1994	1.43	1.85	1.06
Female	High Sugar-Sweetened Beverages Intake	1995	1.48	1.88	1.12
Female	High Sugar-Sweetened Beverages Intake	1996	1.54	1.90	1.20
Female	High Sugar-Sweetened Beverages Intake	1997	1.61	1.94	1.28
Female	High Sugar-Sweetened Beverages Intake	1998	1.68	1.98	1.34
Female	High Sugar-Sweetened Beverages Intake	1999	1.76	2.04	1.42
Female	High Sugar-Sweetened Beverages Intake	2000	1.84	2.09	1.51
Female	High Sugar-Sweetened Beverages Intake	2001	1.94	2.18	1.58
Female	High Sugar-Sweetened Beverages Intake	2002	2.05	2.30	1.69
Female	High Sugar-Sweetened Beverages Intake	2003	2.19	2.46	1.82
Female	High Sugar-Sweetened Beverages Intake	2004	2.35	2.64	1.95
Female	High Sugar-Sweetened Beverages Intake	2005	2.54	2.87	2.11
Female	High Sugar-Sweetened Beverages Intake	2006	2.77	3.10	2.31

Female	High Sugar-Sweetened Beverages Intake	2007	3.04	3.39	2.56
Female	High Sugar-Sweetened Beverages Intake	2008	3.33	3.70	2.85
Female	High Sugar-Sweetened Beverages Intake	2009	3.65	4.07	3.13
Female	High Sugar-Sweetened Beverages Intake	2010	4.00	4.48	3.43
Female	High Sugar-Sweetened Beverages Intake	2011	4.35	4.89	3.72
Female	High Sugar-Sweetened Beverages Intake	2012	4.69	5.32	4.00
Female	High Sugar-Sweetened Beverages Intake	2013	5.03	5.77	4.30
Female	High Sugar-Sweetened Beverages Intake	2014	5.35	6.26	4.47
Female	High Sugar-Sweetened Beverages Intake	2015	5.66	6.81	4.63
Female	High Sugar-Sweetened Beverages Intake	2016	5.94	7.31	4.70
Female	High Sugar-Sweetened Beverages Intake	2017	6.23	7.87	4.77
Female	High Sugar-Sweetened Beverages Intake	2018	6.52	8.43	4.83
Female	High Sugar-Sweetened Beverages Intake	2019	6.81	9.10	4.80
Female	High Sugar-Sweetened Beverages Intake	2020	6.97	9.54	4.74
Female	High Sugar-Sweetened Beverages Intake	2021	7.03	9.92	4.63
Male	High Sugar-Sweetened Beverages Intake	1990	1.20	1.71	0.85
Male	High Sugar-Sweetened Beverages Intake	1991	1.22	1.68	0.88
Male	High Sugar-Sweetened Beverages Intake	1992	1.24	1.67	0.91
Male	High Sugar-Sweetened Beverages Intake	1993	1.27	1.67	0.96
Male	High Sugar-Sweetened Beverages Intake	1994	1.30	1.67	1.01
Male	High Sugar-Sweetened Beverages Intake	1995	1.35	1.69	1.07
Male	High Sugar-Sweetened Beverages Intake	1996	1.41	1.70	1.13
Male	High Sugar-Sweetened Beverages Intake	1997	1.47	1.75	1.20
Male	High Sugar-Sweetened Beverages Intake	1998	1.54	1.79	1.27
Male	High Sugar-Sweetened Beverages Intake	1999	1.61	1.85	1.33

Male	High Sugar-Sweetened Beverages Intake	2000	1.68	1.92	1.38
Male	High Sugar-Sweetened Beverages Intake	2001	1.77	2.00	1.47
Male	High Sugar-Sweetened Beverages Intake	2002	1.87	2.11	1.55
Male	High Sugar-Sweetened Beverages Intake	2003	1.99	2.25	1.64
Male	High Sugar-Sweetened Beverages Intake	2004	2.14	2.41	1.75
Male	High Sugar-Sweetened Beverages Intake	2005	2.31	2.61	1.92
Male	High Sugar-Sweetened Beverages Intake	2006	2.51	2.85	2.12
Male	High Sugar-Sweetened Beverages Intake	2007	2.75	3.12	2.33
Male	High Sugar-Sweetened Beverages Intake	2008	3.01	3.42	2.52
Male	High Sugar-Sweetened Beverages Intake	2009	3.29	3.73	2.78
Male	High Sugar-Sweetened Beverages Intake	2010	3.60	4.04	3.03
Male	High Sugar-Sweetened Beverages Intake	2011	3.90	4.37	3.33
Male	High Sugar-Sweetened Beverages Intake	2012	4.18	4.75	3.58
Male	High Sugar-Sweetened Beverages Intake	2013	4.47	5.16	3.80
Male	High Sugar-Sweetened Beverages Intake	2014	4.75	5.68	3.99
Male	High Sugar-Sweetened Beverages Intake	2015	5.02	6.13	4.07
Male	High Sugar-Sweetened Beverages Intake	2016	5.27	6.63	4.14
Male	High Sugar-Sweetened Beverages Intake	2017	5.54	7.12	4.19
Male	High Sugar-Sweetened Beverages Intake	2018	5.80	7.59	4.22
Male	High Sugar-Sweetened Beverages Intake	2019	6.07	8.18	4.22
Male	High Sugar-Sweetened Beverages Intake	2020	6.22	8.60	4.14
Male	High Sugar-Sweetened Beverages Intake	2021	6.28	8.84	4.04

Sex	Rei name	year	val	upper	lower
Female	Physical Inactivity	1990	11.25	18.78	5.46

Female	Physical Inactivity	1991	11.17	18.53	5.51
Female	Physical Inactivity	1992	11.09	18.44	5.44
Female	Physical Inactivity	1993	11.01	17.87	5.34
Female	Physical Inactivity	1994	10.93	17.21	5.50
Female	Physical Inactivity	1995	10.88	17.00	5.67
Female	Physical Inactivity	1996	10.85	17.26	5.81
Female	Physical Inactivity	1997	10.84	17.27	5.94
Female	Physical Inactivity	1998	10.84	17.10	6.20
Female	Physical Inactivity	1999	10.86	16.86	6.42
Female	Physical Inactivity	2000	10.88	16.67	6.49
Female	Physical Inactivity	2001	10.90	16.99	6.73
Female	Physical Inactivity	2002	10.94	17.02	6.84
Female	Physical Inactivity	2003	11.04	17.10	6.96
Female	Physical Inactivity	2004	11.15	16.91	6.89
Female	Physical Inactivity	2005	11.29	17.53	6.87
Female	Physical Inactivity	2006	11.46	17.71	6.95
Female	Physical Inactivity	2007	11.64	17.80	7.08
Female	Physical Inactivity	2008	11.86	18.23	6.99
Female	Physical Inactivity	2009	12.09	18.54	7.00
Female	Physical Inactivity	2010	12.33	18.72	7.17
Female	Physical Inactivity	2011	12.55	18.91	7.07
Female	Physical Inactivity	2012	12.76	19.43	7.03
Female	Physical Inactivity	2013	12.97	19.98	7.06
Female	Physical Inactivity	2014	13.14	20.57	7.00
Female	Physical Inactivity	2015	13.27	20.88	6.78

Female	Physical Inactivity	2016	13.38	20.89	6.82
Female	Physical Inactivity	2017	13.52	21.13	6.80
Female	Physical Inactivity	2018	13.65	21.36	6.76
Female	Physical Inactivity	2019	13.76	21.30	6.74
Female	Physical Inactivity	2020	13.83	21.68	6.91
Female	Physical Inactivity	2021	13.91	22.04	7.03
Male	Physical Inactivity	1990	4.71	9.82	1.79
Male	Physical Inactivity	1991	4.64	9.80	1.77
Male	Physical Inactivity	1992	4.56	9.67	1.77
Male	Physical Inactivity	1993	4.47	9.45	1.74
Male	Physical Inactivity	1994	4.38	9.31	1.71
Male	Physical Inactivity	1995	4.30	8.95	1.64
Male	Physical Inactivity	1996	4.22	8.49	1.57
Male	Physical Inactivity	1997	4.15	8.27	1.55
Male	Physical Inactivity	1998	4.09	7.95	1.59
Male	Physical Inactivity	1999	4.03	7.64	1.67
Male	Physical Inactivity	2000	3.99	7.37	1.61
Male	Physical Inactivity	2001	3.96	7.19	1.59
Male	Physical Inactivity	2002	3.94	6.96	1.61
Male	Physical Inactivity	2003	3.96	6.91	1.70
Male	Physical Inactivity	2004	4.00	6.87	1.67
Male	Physical Inactivity	2005	4.05	7.08	1.70
Male	Physical Inactivity	2006	4.11	7.20	1.79
Male	Physical Inactivity	2007	4.19	7.40	1.77
Male	Physical Inactivity	2008	4.29	7.64	1.83

Male	Physical Inactivity	2009	4.41	7.85	1.83
Male	Physical Inactivity	2010	4.55	8.22	1.81
Male	Physical Inactivity	2011	4.66	8.58	1.78
Male	Physical Inactivity	2012	4.80	8.96	1.82
Male	Physical Inactivity	2013	4.95	9.10	1.85
Male	Physical Inactivity	2014	5.13	9.28	1.92
Male	Physical Inactivity	2015	5.30	9.58	1.97
Male	Physical Inactivity	2016	5.47	9.99	2.05
Male	Physical Inactivity	2017	5.68	10.64	2.24
Male	Physical Inactivity	2018	5.89	10.85	2.29
Male	Physical Inactivity	2019	6.08	11.43	2.26
Male	Physical Inactivity	2020	6.24	11.84	2.34
Male	Physical Inactivity	2021	6.40	12.17	2.39

Sex	Rei name	year	val	upper	lower
Female	Chewing Tobacco	1990	0.37	0.69	0.17
Female	Chewing Tobacco	1991	0.37	0.69	0.16
Female	Chewing Tobacco	1992	0.37	0.67	0.17
Female	Chewing Tobacco	1993	0.37	0.66	0.18
Female	Chewing Tobacco	1994	0.37	0.65	0.18
Female	Chewing Tobacco	1995	0.37	0.67	0.17
Female	Chewing Tobacco	1996	0.37	0.67	0.17
Female	Chewing Tobacco	1997	0.37	0.68	0.17
Female	Chewing Tobacco	1998	0.37	0.67	0.17
Female	Chewing Tobacco	1999	0.37	0.69	0.17

Female	Chewing Tobacco	2000	0.37	0.68	0.17
Female	Chewing Tobacco	2001	0.37	0.69	0.17
Female	Chewing Tobacco	2002	0.37	0.68	0.18
Female	Chewing Tobacco	2003	0.37	0.69	0.18
Female	Chewing Tobacco	2004	0.37	0.69	0.17
Female	Chewing Tobacco	2005	0.37	0.69	0.17
Female	Chewing Tobacco	2006	0.37	0.68	0.17
Female	Chewing Tobacco	2007	0.37	0.68	0.17
Female	Chewing Tobacco	2008	0.37	0.69	0.17
Female	Chewing Tobacco	2009	0.37	0.70	0.18
Female	Chewing Tobacco	2010	0.38	0.73	0.18
Female	Chewing Tobacco	2011	0.40	0.76	0.19
Female	Chewing Tobacco	2012	0.41	0.79	0.20
Female	Chewing Tobacco	2013	0.42	0.82	0.21
Female	Chewing Tobacco	2014	0.44	0.84	0.21
Female	Chewing Tobacco	2015	0.45	0.85	0.21
Female	Chewing Tobacco	2016	0.46	0.88	0.22
Female	Chewing Tobacco	2017	0.48	0.91	0.23
Female	Chewing Tobacco	2018	0.49	0.95	0.23
Female	Chewing Tobacco	2019	0.49	0.96	0.23
Female	Chewing Tobacco	2020	0.49	0.95	0.23
Female	Chewing Tobacco	2021	0.49	0.94	0.23
Male	Chewing Tobacco	1990	0.96	1.57	0.55
Male	Chewing Tobacco	1991	0.97	1.57	0.55
Male	Chewing Tobacco	1992	0.97	1.56	0.55

Male	Chewing Tobacco	1993	0.96	1.59	0.55
Male	Chewing Tobacco	1994	0.96	1.60	0.55
Male	Chewing Tobacco	1995	0.95	1.64	0.54
Male	Chewing Tobacco	1996	0.95	1.64	0.54
Male	Chewing Tobacco	1997	0.95	1.61	0.54
Male	Chewing Tobacco	1998	0.95	1.63	0.54
Male	Chewing Tobacco	1999	0.96	1.62	0.55
Male	Chewing Tobacco	2000	0.96	1.64	0.54
Male	Chewing Tobacco	2001	0.96	1.64	0.54
Male	Chewing Tobacco	2002	0.97	1.67	0.54
Male	Chewing Tobacco	2003	0.97	1.69	0.54
Male	Chewing Tobacco	2004	0.97	1.69	0.54
Male	Chewing Tobacco	2005	0.97	1.67	0.54
Male	Chewing Tobacco	2006	0.97	1.66	0.53
Male	Chewing Tobacco	2007	0.97	1.69	0.53
Male	Chewing Tobacco	2008	0.97	1.68	0.54
Male	Chewing Tobacco	2009	0.97	1.66	0.53
Male	Chewing Tobacco	2010	0.99	1.68	0.54
Male	Chewing Tobacco	2011	1.02	1.71	0.55
Male	Chewing Tobacco	2012	1.04	1.74	0.56
Male	Chewing Tobacco	2013	1.06	1.80	0.58
Male	Chewing Tobacco	2014	1.08	1.83	0.59
Male	Chewing Tobacco	2015	1.10	1.82	0.62
Male	Chewing Tobacco	2016	1.12	1.84	0.62
Male	Chewing Tobacco	2017	1.14	1.88	0.64

Male	Chewing Tobacco	2018	1.16	1.90	0.65
Male	Chewing Tobacco	2019	1.16	1.91	0.66
Male	Chewing Tobacco	2020	1.16	1.90	0.67
Male	Chewing Tobacco	2021	1.16	1.90	0.66

Sex	Rei name	year	val	upper	lower
Female	Kidney Dysfunction	1990	0.87	1.30	0.63
Female	Kidney Dysfunction	1991	0.87	1.29	0.62
Female	Kidney Dysfunction	1992	0.86	1.28	0.61
Female	Kidney Dysfunction	1993	0.85	1.27	0.60
Female	Kidney Dysfunction	1994	0.84	1.26	0.59
Female	Kidney Dysfunction	1995	0.83	1.25	0.59
Female	Kidney Dysfunction	1996	0.82	1.24	0.58
Female	Kidney Dysfunction	1997	0.81	1.22	0.57
Female	Kidney Dysfunction	1998	0.80	1.21	0.57
Female	Kidney Dysfunction	1999	0.80	1.19	0.56
Female	Kidney Dysfunction	2000	0.80	1.19	0.56
Female	Kidney Dysfunction	2001	0.80	1.20	0.57
Female	Kidney Dysfunction	2002	0.81	1.22	0.57
Female	Kidney Dysfunction	2003	0.82	1.23	0.57
Female	Kidney Dysfunction	2004	0.82	1.23	0.58
Female	Kidney Dysfunction	2005	0.82	1.22	0.57
Female	Kidney Dysfunction	2006	0.81	1.22	0.56
Female	Kidney Dysfunction	2007	0.80	1.20	0.55
Female	Kidney Dysfunction	2008	0.78	1.19	0.54

Female	Kidney Dysfunction	2009	0.77	1.17	0.53
Female	Kidney Dysfunction	2010	0.76	1.16	0.52
Female	Kidney Dysfunction	2011	0.75	1.15	0.52
Female	Kidney Dysfunction	2012	0.76	1.17	0.52
Female	Kidney Dysfunction	2013	0.76	1.19	0.52
Female	Kidney Dysfunction	2014	0.77	1.21	0.52
Female	Kidney Dysfunction	2015	0.77	1.21	0.52
Female	Kidney Dysfunction	2016	0.76	1.19	0.51
Female	Kidney Dysfunction	2017	0.74	1.15	0.49
Female	Kidney Dysfunction	2018	0.72	1.12	0.48
Female	Kidney Dysfunction	2019	0.71	1.11	0.47
Female	Kidney Dysfunction	2020	0.72	1.12	0.48
Female	Kidney Dysfunction	2021	0.73	1.14	0.49
Male	Kidney Dysfunction	1990	0.77	1.14	0.55
Male	Kidney Dysfunction	1991	0.76	1.13	0.54
Male	Kidney Dysfunction	1992	0.74	1.11	0.53
Male	Kidney Dysfunction	1993	0.73	1.10	0.52
Male	Kidney Dysfunction	1994	0.72	1.09	0.51
Male	Kidney Dysfunction	1995	0.72	1.08	0.51
Male	Kidney Dysfunction	1996	0.71	1.07	0.50
Male	Kidney Dysfunction	1997	0.70	1.06	0.49
Male	Kidney Dysfunction	1998	0.69	1.04	0.49
Male	Kidney Dysfunction	1999	0.69	1.04	0.49
Male	Kidney Dysfunction	2000	0.69	1.04	0.48
Male	Kidney Dysfunction	2001	0.70	1.05	0.49

Male	Kidney Dysfunction	2002	0.70	1.06	0.49
Male	Kidney Dysfunction	2003	0.71	1.07	0.50
Male	Kidney Dysfunction	2004	0.71	1.07	0.50
Male	Kidney Dysfunction	2005	0.71	1.07	0.50
Male	Kidney Dysfunction	2006	0.71	1.06	0.49
Male	Kidney Dysfunction	2007	0.70	1.05	0.49
Male	Kidney Dysfunction	2008	0.69	1.04	0.48
Male	Kidney Dysfunction	2009	0.68	1.03	0.47
Male	Kidney Dysfunction	2010	0.67	1.02	0.47
Male	Kidney Dysfunction	2011	0.67	1.02	0.46
Male	Kidney Dysfunction	2012	0.67	1.03	0.46
Male	Kidney Dysfunction	2013	0.67	1.04	0.46
Male	Kidney Dysfunction	2014	0.68	1.05	0.46
Male	Kidney Dysfunction	2015	0.68	1.06	0.45
Male	Kidney Dysfunction	2016	0.67	1.04	0.45
Male	Kidney Dysfunction	2017	0.65	1.01	0.44
Male	Kidney Dysfunction	2018	0.63	0.99	0.43
Male	Kidney Dysfunction	2019	0.63	0.98	0.42
Male	Kidney Dysfunction	2020	0.63	0.99	0.43
Male	Kidney Dysfunction	2021	0.65	1.00	0.44
